# Systemic proteomics and miRNA profile analysis of exosomes derived from human pluripotent stem cells

**DOI:** 10.1186/s13287-022-03142-1

**Published:** 2022-09-05

**Authors:** Youkun Bi, Xinlong Qiao, Qun Liu, Shaole Song, Keqi Zhu, Xun Qiu, Xiang Zhang, Ce jia, Huiwen Wang, Zhiguang Yang, Ying Zhang, Guangju Ji

**Affiliations:** 1 Key Laboratory of Interdisciplinary Research, Institute of Biophysics, Chinese Academy of Sciences, Beijing, 100101 China; 2 University of Chinese Academy of Sciences, Beijing, 100049 China; 3 Department of Medical Oncology, The Second Affiliated Hospital of Dalian Medical University, Dalian, 116023 China; 4 Sixth Department of Liver Disease, Dalian Public Health Clinical Center, Dalian Medical University, Dalian, 116023 China

**Keywords:** Human embryonic stem cells, Human-induced pluripotent stem cells, Human umbilical cord mesenchymal stem cells, Exosomes, Proteomics, miRNA

## Abstract

**Background:**

Increasing studies have reported the therapeutic effect of mesenchymal stem cell (MSC)-derived exosomes by which protein and miRNA are clearly characterized. However, the proteomics and miRNA profiles of exosomes derived from human embryonic stem cells (hESCs) and human-induced pluripotent stem cells (hiPSCs) remain unclear.

**Methods:**

In this study, we isolated exosomes from hESCs, hiPSCs, and human umbilical cord mesenchymal stem cells (hUC-MSCs) via classic ultracentrifugation and a 0.22-μm filter, followed by the conservative identification. Tandem mass tag labeling and label-free relative peptide quantification together defined their proteomics. High-throughput sequencing was performed to determine miRNA profiles. Then, we conducted a bioinformatics analysis to identify the dominant biological processes and pathways modulated by exosome cargos. Finally, the western blot and RT-qPCR were performed to detect the actual loads of proteins and miRNAs in three types of exosomes.

**Results:**

Based on our study, the cargos from three types of exosomes contribute to sophisticated biological processes. In comparison, hESC exosomes (hESC-Exos) were superior in regulating development, metabolism, and anti-aging, and hiPSC exosomes (hiPSC-Exos) had similar biological functions as hESC-Exos, whereas hUC-MSCs exosomes (hUC-MSC-Exos) contributed more to immune regulation.

**Conclusions:**

The data presented in our study help define the protein and miRNA landscapes of three exosomes, predict their biological functions via systematic and comprehensive network analysis at the system level, and reveal their respective potential applications in different fields so as to optimize exosome selection in preclinical and clinical trials.

**Supplementary Information:**

The online version contains supplementary material available at 10.1186/s13287-022-03142-1.

## Introduction

Extracellular vesicles (EVs) are tiny vesicles actively secreted by cells, mainly containing exosomes, microvesicles (MVs), and apoptotic bodies [40, 56, 57]. They are widely distributed in multiple body fluids, such as saliva, breast milk, blood, cerebrospinal fluid, bile, and urine [57]. Among them, exosomes have a lipid bilayer with a diameter of 40–200 nm, a buoyant density of 1.13–1.18 g/ml in the sucrose gradient, and a cup-shaped appearance under an electron microscope [21, 53]. Various bioactive compounds, including proteins, nucleic acids, and lipids, endow the intercellular communication function of exosomes between cells [16, 17]. The lipid bilayer is a delicate barrier that protects the contents of exosomes from body fluid enzymatic degeneration [49]. Stable exosomes can mediate complicated physiological and pathological processes via paracrine activity, including organ and reproductive development, antigen presentation, neuronal communication, immune response, aging regulation, and cell proliferation [57, 62].

Exosome formation and release is a finely regulated process, and the sorting of exosome-encapsulated contents occurs by virtue of the mobilization of various proteins [22, 47]. Multiple proteins are crucial to exosome formation, which contains the endosomal sorting complex required for transport (ESCRT), tetraspanins (CD9, CD63, and CD81), apoptosis-linked gene 2-interactin protein X (Alix), and tumor susceptibility gene 101 (TSG101) [38, 55]. The intracellular trafficking of exosomes is driven by the collective activity of a series of proteins, including molecular switch RAB GTPases and cytoskeletal proteins, such as actin and tubulin [24]. Subsequently, exosome secretion is complemented by the SNARE complex and synaptotagmin family [22]. Research has determined that the budding and shedding of exosomes rely on calcium activity and ESCRT recruitment [15]. As a messenger, exosome release is involved in cell crosstalk in response to cellular physiology and pathological changes, such as activation, pH change, hypoxia, radiation damage, or cell stress [61].

To uncover the components of exosomes and their possible physiological and pathological functions, proteomic, transcriptomic, and other techniques are often used to study exosome RNAs and proteins from different species, tissues, and cells [44]. Exosome proteomics analysis typically includes three steps: the isolation, purification, and characterization of exosomes, the identification of protein components using mass spectrometry, and raw data analysis [14]. The cellular state significantly affects the protein composition and abundance of exosomes. At present, quantitative proteomic techniques for analyzing protein characterization and abundance are used to elucidate the mechanism underlying exosome production and deepen our understanding of the physiological and pathological roles of exosomes in cells [39]. Among them, mass spectrometry-based quantitative proteomics technologies are widely used, mainly including label-free and stable isotope-labeled methods [13, 42]. miRNAs are small bioactive molecules that are closely associated with various life activities. The high-throughput sequencing technique is characterized by high sensitivity and precision and has wide application in miRNA sequencing (18–30 nt) [6]. Given the development of current technology, there is no barrier to dissecting the protein and miRNA profiles of exosomes.

The hESCs and hiPSCs have high differentiation potential [36]. However, their direct application in treatment is limited due to the risk of malignancy and ethical issues [31]. In contrast, mesenchymal stem cells (MSCs) have a broader application for intervention in multiple diseases in clinical settings [8]. However, their direct use still faces several limitations, including a low survival rate, immunological rejection, and safety issues [8, 43]. Currently, much attention has been paid to exosomes because of their biosecurity, stability, non-aneuploidy, and low immunogenicity [51]. Previous studies have documented the component comparison of MSCs derived from different tissues and their exosomes containing proteomic and miRNA profiles [59]. Gong et al. also depicted the regulatory network of hESC extracellular vesicles (EVs) in terms of the proteome, but only in aging-related pathways without focusing on others [19]. Questa et al. weaved the EV proteomic atlas of hiPSCs derived from human foreskin fibroblasts (HFFs) [45]. Although these studies have partly reported the protein components of EVs, they did not focus solely on exosomes, and their comprehensive proteomic analysis is not clearly articulated. In particular, the miRNA profiles have not yet been described. To address this, exosomes isolated from hESCs, hiPSCs, and hUC-MSCs were selected in the present study for further investigation targeting their proteome and miRNA profiles to gain novel insights for their research and clinical application.

## Methods

### Preparation and characterization of exosomes

hESCs and hiPSCs were cultured in ncTarget medium (cat. no. RP01020; Nuwacell. Ltd, China), while the 3^rd^ generation of hUC-MSCs were cultured in serum-free ncMission hMSC medium (cat. no. RP02010; Nuwacell. Ltd, China). Next, 350 mL of the supernatant of cells in the logarithmic growth phase (~ 80% confluency) was collected for exosome purification. The exosomes were extracted in accordance with a series of recognized centrifugation and ultracentrifugation, as previously described [34, 52]. Briefly, the conditioned media (CM) were collected and subjected to gradient centrifugation (300 × *g* for 10 min, 2000 × *g* for 15 min, 10, 000 × *g* for 30 min) to separate cell debris. Exosomes were pelleted from the collected supernatants at 100, 000 × *g* for 70 min using a Ti45 rotor (Beckman Coulter, USA). They were then resuspended in PBS for the next filtration using a 0.22-μm MF-Millipore^™^ Membrane filter (Sigma, USA). The filtrate was subjected to ultracentrifugation at 100, 000 × *g* for 70 min. The final exosome pellet was resuspended in PBS for the next analysis. Dynamic light scattering (DLS) system (Wyatt Technology, USA) was used to measure the concentration and size of the isolated exosomes.

### Transmission electron microscopy (TEM)

TEM scanning was performed to describe the morphology of isolated exosomes, as previously described. The resuspended exosomes (1 μg in 10 μl of PBS) were planted on a carbon-coated copper grid (200-mesh) and allowed to adsorb onto it for 2 min, followed by two washes with double-distilled water. Then, the grids were negatively stained with 8 μL of 2% uranyl acetate solution for 1 min. After natural drying, the samples were examined using a Tecnai Spirit system (Thermo Fisher, USA) at 120 kV.

### Dynamic light scattering

The size distribution of exosomes was described via nanoparticle tracking analysis using a dynamic light scatterometer (Wyatt Technology, USA) according to the manufacturer’s instructions (271-DPN), as previously reported[23]. Briefly, the exosome pellet was resuspended in 100 μL of PBS. Then, 50 μL of the above exosomes was added to 1450 μL of PBS and vortexed for 30 s. Exosomes (1.5 mL) were transferred to a disposable cuvette for equilibration at 25 °C for 30 s. The dispersant refractive index value was 1.37, and both Z-average and polydispersity (PDI) determined the size of the exosome particles. Three independent measurements were taken for each sample.

### Tandem mass tag (TMT) labeling and label-free relative peptide quantification (LFQ) analysis

Total protein was extracted from exosomes, and the concentration was quantified using the Bradford method. The samples were separated by 12.5% sodium dodecyl sulfate polyacrylamide gel electrophoresis (SDS-PAGE). The protein suspensions were digested with trypsin (cat. no. 9002-07-7; Sigma, USA) in 40 μL of tetraethylammonium bromide (TEAB) buffer for 12 h at 37 °C and then labeled with TMTsixplex^™^ isobaric label reagent set (cat. no. 90061; Thermo Fisher, USA). TMT-labeled peptides were fractionated by reversed-phase chromatography using an Agilent 1260 Infinity II HPLC system (Agilent Technology, USA). LC-MSC/MS analysis was performed by LC-Bio Technology Co., Ltd. (Hangzhou, China). Briefly, the analysis was completed using the combination of a Q Exactive Plus mass spectrometer and Easy nLC (Thermo Fisher, USA). Survey scans were acquired at a resolution of 70,000 at *m/z* 200. MS/MS spectra were captured by the MASCOT engine using the built-in software Proteome Discoverer 2.4. Label-free relative peptide quantification analysis was also performed by LC-Bio Technology Co., Ltd. as previously reported [27]. Briefly, the digested proteins of exosomes were pre-separated by HPLC and detected by mass spectrometry. Raw files from technical and biological replicates were filtered, de novo sequenced, and assigned with protein ID using PEAKS 8.0 by searching against the human Swiss-Prot database. Three independent exosome samples were subjected to TMT or label-free assay. The final protein profiles of exosomes were determined by overlapping the data of TMT (mascot score > 60, abundance > 100, and *P* < 0.05) and label-free (at least two tests results > 0 and *P* < 0.05).

Differentially expressed proteins (DEPs) were considered valid after the data were normalized by protein loading and differential *p*-value false discovery rates (FDR) corrected [30]. In particular, the proteins with log_2_|fold change|> 1 (|log_2_FC|> 1), as well as statistical significance (*P* < 0.05), were classified as enriched in exosomes.

### miRNA profile analysis

Total RNA was extracted from isolated exosomes using the *mirVana*^™^ miRNA Isolation Kit (cat. no. AM1560; Thermo Fisher, USA). The RNA samples were subjected to quality inspection for the miRNA microarray assay performed by LC-Bio Technology Co., Ltd. The analysis of raw data was performed as previously reported [9]. Differentially expressed miRNAs were selected as candidate miRNAs at a *p*-value < 0.05 and |log_2_FC|> 2. Data were omitted if they corresponded to questionable miRNAs, according to previous reports or in-house validated miRNAs [12]. The miRNA target genes were determined by the overlap of prediction of Targetscan (https://www.targetscan.org/vert_80/) and miRanda database (http://www.bioinformatics.com.cn/l) under the restriction of threshold value > 90 (Targetscan) and maximum free energy < − 10 (miRanda).

### Bioinformatics analysis

Raw data were subjected to analysis using the Maxquant software package (v1.6.0), and the Swiss-Prot_Human data was set as a reference (20,600 proteins) (Proteome ID: UP000005640). Identified proteins were mapped to Gene Ontology (GO) and Kyoto Encyclopedia of Genes and Genomes (KEGG) based on KOBAS analysis [60] (http://kobas.cbi.pku.edu.cn/) terms to determine their biological and functional properties. Cytoscape software and igraph package in R software (version 3.6.1) were used to draw the interwoven network of exosome proteins or miRNAs between signaling pathways.

### Statistical analysis

All experiments were performed in triplicate. The quantitative repeatability of proteins and miRNAs was elevated via principal component analysis (PCA), relative standard deviation, and Pearson’s correlation coefficient. The results were analyzed using unpaired Student’s *t-*test. Data are expressed as the mean ± standard error of the mean (SEM). *P-*values were considered statistically significant at *P < 0.05, **P < 0.01, and ***P < 0.001.

## Results

### Exosome isolation and identification

**Fig. 1:**
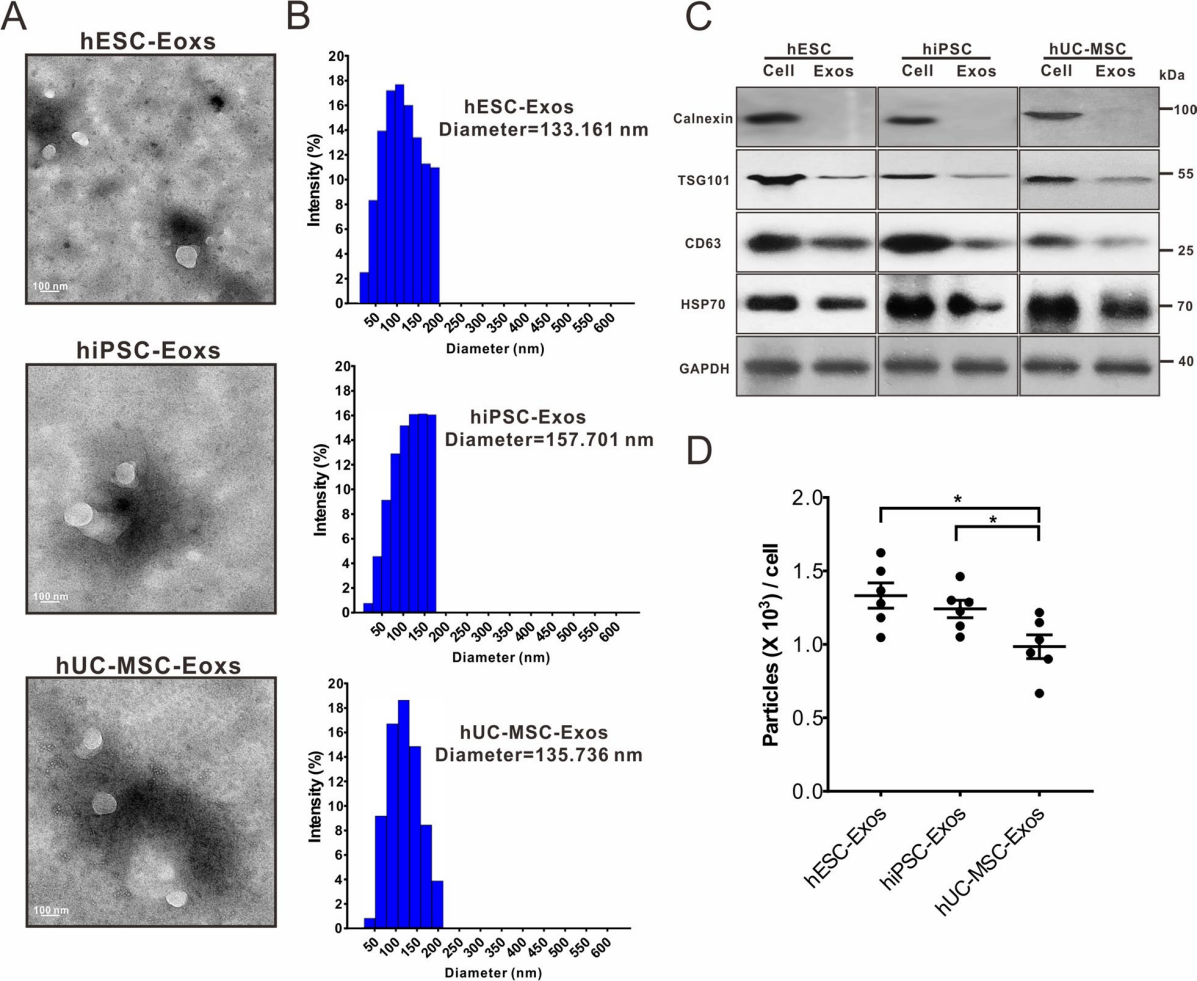
Quality analysis of isolated exosomes. **A** Representative TEM micrograph of exosomes derived from hESCs, hiPSCs, and hUC-MSCs. Scale bar = 100 nm. **B** DLS system describing the diameter of isolated exosomes. **C** Western blot assay determining the expression of Calnexin, TSG101, CD63, and HSP70 in cells and exosomes. GAPDH was set as the internal reference. **D** Evaluation of mean exosomes yield per cell. All statistical data are presented as means ± standard deviation of two-tailed unpaired Student’s *t*-tests. ^*^P < 0.05

The culture and identification of three human pluripotent stem cells were described in Additional file 1. Exosomes were isolated from the conditioned media of three types of stem cells (Additional file 1: Fig. S1) and identified based on morphology, particle size, concentration, and surface markers [19]. TEM revealed that these exosomes had a cup-shaped morphology (Fig. 1A), and DLS revealed that their size distribution was within 50–200 nm (Fig. 1B). Western blotting indicated that the isolated exosomes carried the positive markers CD63, TSG101, and HSP70 but not the negative marker calnexin (Fig. 1C). Each hESC had a similar exosome yield to that of hiPSCs, and both were higher than that of hUC-MSCs (Fig. 1D). These results indicate that representative exosomes were arrested, with no differences in shape; however, differences were found among the concentrations of exosomes isolated from the three types of stem cells.

### Bioinformatics of shared and top-loaded proteins

The SDS-PAGE and quantitative analyses revealed that while the protein concentrations of hESC-Exos and hiPSC-Exos were not different, they were both higher than those of hUC-MSC-Exos (Additional file 1: Fig. S2A and B). The PCA plot indicated that exosomes had good uniformity within each group (Additional file 1: Fig. S2C). The peptide fragments digested via enzymatic hydrolysis passed the quality inspection (Additional file 1: Fig. S2D–G) and then were subjected to next bioinformatics analysis. Moreover, the proteins of three exosomes were mainly located in the extracellular region, followed by the cytoplasm (Additional file 1: Fig. S2H). To describe the protein profiles of the three types of exosomes, we integrated the TMT (Additional file 2) and label-free data (Additional file 3) under the conditions of mascot score > 60 and abundance > 100 (TMT assay) and abundance of at least two repetitions > 0 (label-free assay). Finally, 554, 437, and 911 seeds were selected for analyzing the protein profiles of hESC-Exos, hiPSC-Exos, and hUC-MSC-Exos, respectively (Additional file 1: Fig. S3A). Then these candidates were subjected to Venn diagram assay to select shared proteins (Additional file 1: Fig. S3B). Bioinformatics analysis revealed the 303 shared proteins dominated the regulation in these signaling pathways including regulation of actin cytoskeleton, focal adhesion, PI3K-AKT, carbon metabolism, etc. (Additional file 1: Fig. S3C). GO analysis also indicated that the shared proteins were enriched in extracellular exosome, membrane, protein binding, and extracellular region, (Additional file 1: Fig. S3D).

**Fig. 2:**
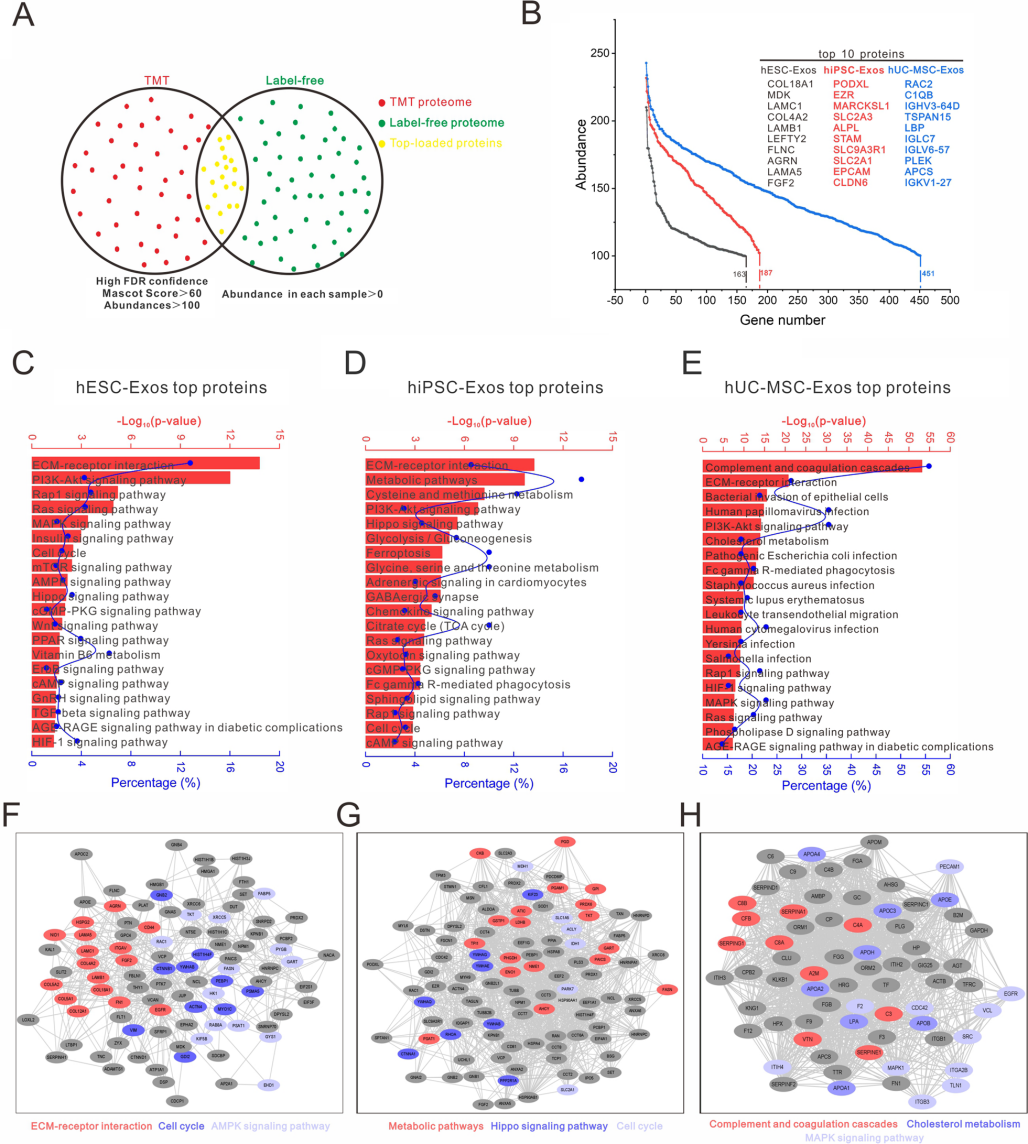
The top-loaded proteins in exosomes were involved in sophisticated network regulation. **A** Screening of the top-expressed candidate proteins from TMT and label-free protein pools under the conditions of high FDR confidence, mascot score > 60, and abundance > 100 (red dots) and abundance in each sample > 0 (green dots). Yellow dots represent the top-loaded proteins of exosomes. **B** Expression abundance curve of the three exosome types and the top ten loaded proteins in exosomes. **C**–**E** KEGG analysis of the top-expressed proteins in hESC-Exos, hiPSC-Exos, and hUC-MSC-Exos, respectively, ranked from high to low -Log_10_(*P*-value). The red bar represents the -Log_10_ (*P*-value) and the blue dot the proportion of candidate genes in the total pathway-related gene pool. **F**–**H** PPI analysis of top-expressed proteins in hESC-Exos, hiPSC-Exos, and hUC-MSC-Exos, respectively, according to the KOBAS algorithm. The color of the histogram corresponds to the color of the gene clusters in the PPI network

Furthermore, the top-loaded proteins were selected from the candidates with high FDR confidence (TMT assay) and abundance of each repetition > 0 (label-free assay) (Fig. 2A). Based on these results, 163, 187, and 451 proteins were screened in the hESC-Exos, hiPSC-Exos, and hUC-MSC-Exos, respectively (Additional file 1: Fig. S3E). We then described the expression abundance curve of the three types of exosomes and identified the top ten gene symbols of loaded proteins in exosomes (Fig. 2B). The highly loaded proteins were subjected to bioinformatics based on the KOBAS algorithm. The proteomes of the three types of exosomes were all involved in a complex signaling pathway regulatory network, and the top 20 pathways were then sorted based on the value of -log_10_ (*P*-value) (Fig. 2C–E). All proteomes were enriched in the extracellular matrix (ECM)-receptor interaction and PI3K-AKT signaling pathways. The protein–protein interaction (PPI) analysis revealed that the top-loaded proteins in both hESC-Exos and hiPSC-Exos were enriched in cell cycle and metabolic pathway, whereas the top-loaded proteins in hUC-MSC-Exos were enriched in immunity regulation-related pathways (Fig. 2F–H).

### Pairwise comparison of exosome proteomics

**Fig. 3:**
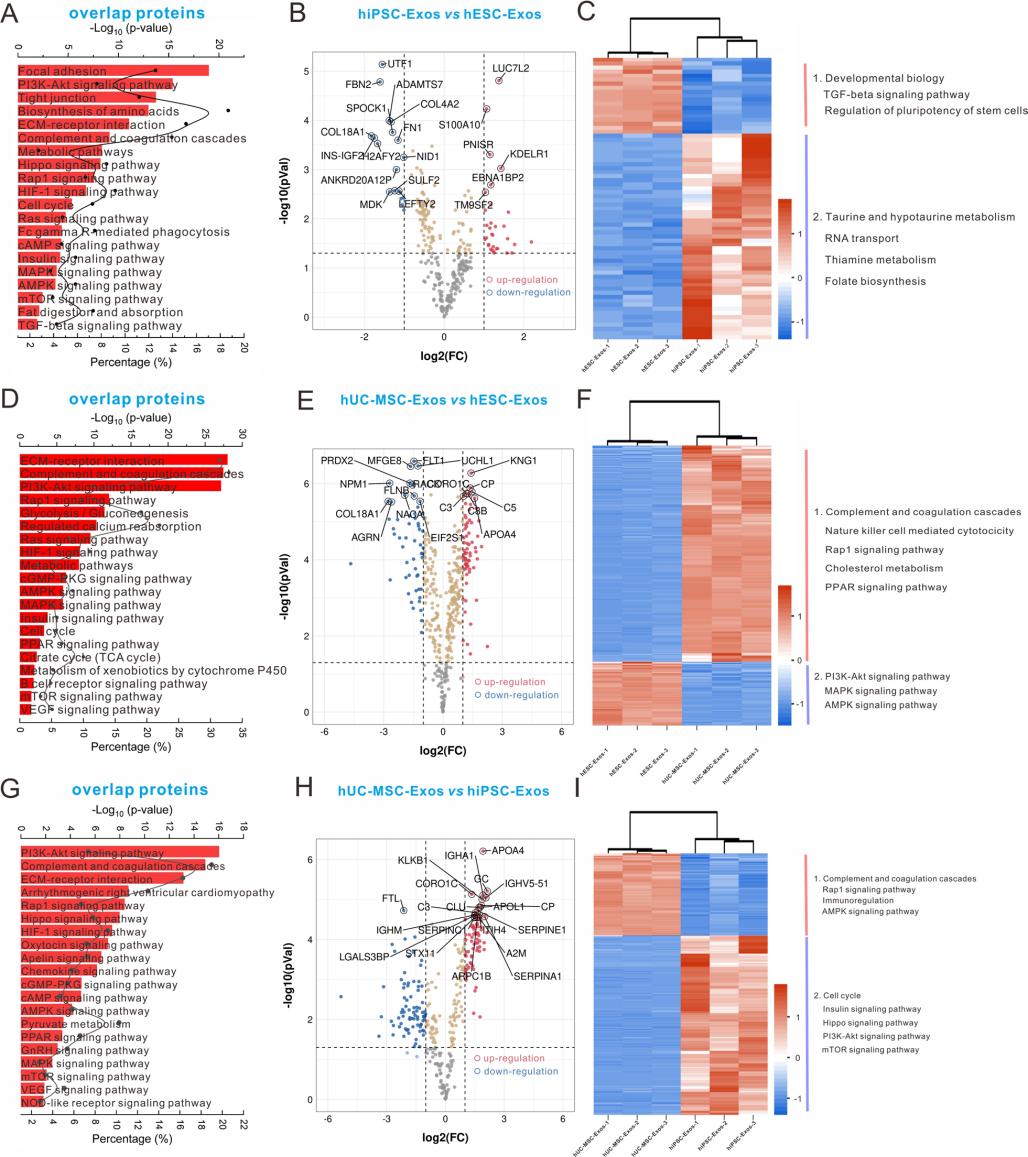
The shared proteins had different signaling regulation abilities when performing pairwise bioinformatics analysis. **A**, **D**, and **G** KEGG analyses of overlapping proteins in hESC-Exos and hiPSC-Exos **A**, hESC-Exos and hUC-MSC-Exos **B**, and hiPSC-Exos and hUC-MSC-Exos **G**. The red bar and black dot represent the -Log_10_ (*P*-value) and the proportion of candidate genes in the total pathway-related gene pool, respectively. **B**, **E**, and **H** Volcano diagrams of differentially expressed proteins in hESC-Exos *vs* hiPSC-Exos **B**, hESC-Exos *vs* hUC-MSC-Exos **E**, and hiPSC-Exos *vs* hUC-MSC-Exos **E** at *P* < 0.05 and |log_2_FC|≥ 1. Histogram of the GO and KEGG analyses of upregulated protein clusters ranked from high to low significance. **C**, **F**, and **I** Heatmap of the levels of shared proteins between hESC-Exos and hiPSC-Exos **C**, hESC-Exos and hUC-MSC-Exos **F**, and hiPSC-Exos and hUC-MSC-Exos **I**. The significantly enriched pathways are shown on the right

To evaluate the difference between each two exosome proteomes, we constructed a Venn diagram to recognize the unique and overlapping protein-coding gene clusters. The shared protein-coding genes were then subjected to volcano and heatmap analysis and to GSEA, and differentially expressed proteins were filtered at *P* < 0.05 and |log_2_FC|≥ 1. Comparison of hESC-Exos and hiPSC-Exos (Additional file 1: Fig. S4A) revealed that their unique clusters were both enriched in ECM-receptor interaction and complement and coagulation cascades (Additional file 1: Fig. S4B and C) while overlapping clusters participated in multiple signaling pathways, including metabolic, cell cycle, and Hippo pathways (Fig. 3A). Significant differentially expressed protein-coding genes are presented in the volcano plot (Fig. 3 B). Heatmap analysis revealed that Cluster 1 proteins related to developmental biology, TGF-β signaling, and pluripotency regulation were enriched in hESC-Exos. Proteins in Cluster 2, which were mainly enriched in hiPSC-Exos, were related to taurine and hypotaurine metabolism, RNA transport, thiamine metabolism, and folate biosynthesis (Fig. 3 C). GSEA further emphasized the more important role of hESC-Exos in developmental biology and cell cycle compared to hiPSC-Exos (Additional file 1: Fig. S5).

The comparison between hESC-Exos and hUC-MSC-Exos (Additional file 1: Fig. S4 D) revealed that unique protein-coding gene clusters of hESC-Exos were mainly involved in EGFR, TGF-*β*, cell cycle, pluripotency regulation, and Wnt signaling (Additional file 1: Fig. S4E). In contrast, hUC-MSC-Exos unique clusters were enriched in many immunoregulation-related signaling pathways such as complement system, inflammation regulation, and microbial infection (Additional file 1: Fig. S4F). Overlapping clusters were highly enriched in ECM-receptor interaction, complement and coagulation cascades, and PI3K-AKT signaling (Fig. 3D). The volcano plot depicts the differences between hESC-Exos and hUC-MSC-Exos (Fig. 3E). The upregulated proteins in hUC-MSC-Exos (Cluster 1) were mainly involved in the complement response, natural killer cell activity, and Rap1 and PPAR signaling. In contrast, hESC-Exos upregulated proteins were mainly involved in PI3K-AKT, MAPK, and AMPK signaling (Fig. 3F). GSEA confirmed the higher regulatory ability of hiPSC-Exos in pluripotency (Additional file 1: Fig. S6A and D) and that of hESC-Exos in immunoregulation, including natural killer cell-mediated cytotoxicity (Additional file 1: Fig. S6B and E) and regulation of COVID19-SARS-CoV_2_ (Additional file 1: Fig. S6C and F).

The hiPSC-Exos and hUC-MSC-Exos comparison (Additional file 1: Fig. S4G) revealed that the unique hiPSC-Exos protein-coding gene set was significantly associated with non-homologous end-joining, TGF-β, and vitamin B6 metabolism (Additional file 1: Fig. S4H), whereas that of hUC-MSC-Exos was mainly involved in immunoregulation-related pathways (Additional file 1: Fig. S4I). Their overlapping proteins also participated in the regulation of ECM-receptor interactions, complement and coagulation cascades, and PI3K-AKT signaling (Fig. 3G). The volcano plot presents the differentially expressed proteins (Fig. 3H). Cluster 1 proteins in the heatmap indicated that hUC-MSC-Exos mainly regulated the immune response and the AMPK signaling pathway while hiPSC-Exos mainly regulated the cell cycle and insulin, Hippo, and mTOR signaling pathways (Fig. 3I). GSEA further supported the dominant role of hiPSC-Exos in the regulation of developmental processes (Additional file 1: Fig. S7A and D) and maintenance of pluripotency (Additional file 1: Fig. S7B and E), whereas hUC-MSC-Exos were mostly involved in the immunoregulation process (Additional file 1: Fig. S7C and F).

### Bioinformatics of overlapping proteomes

**Fig. 4:**
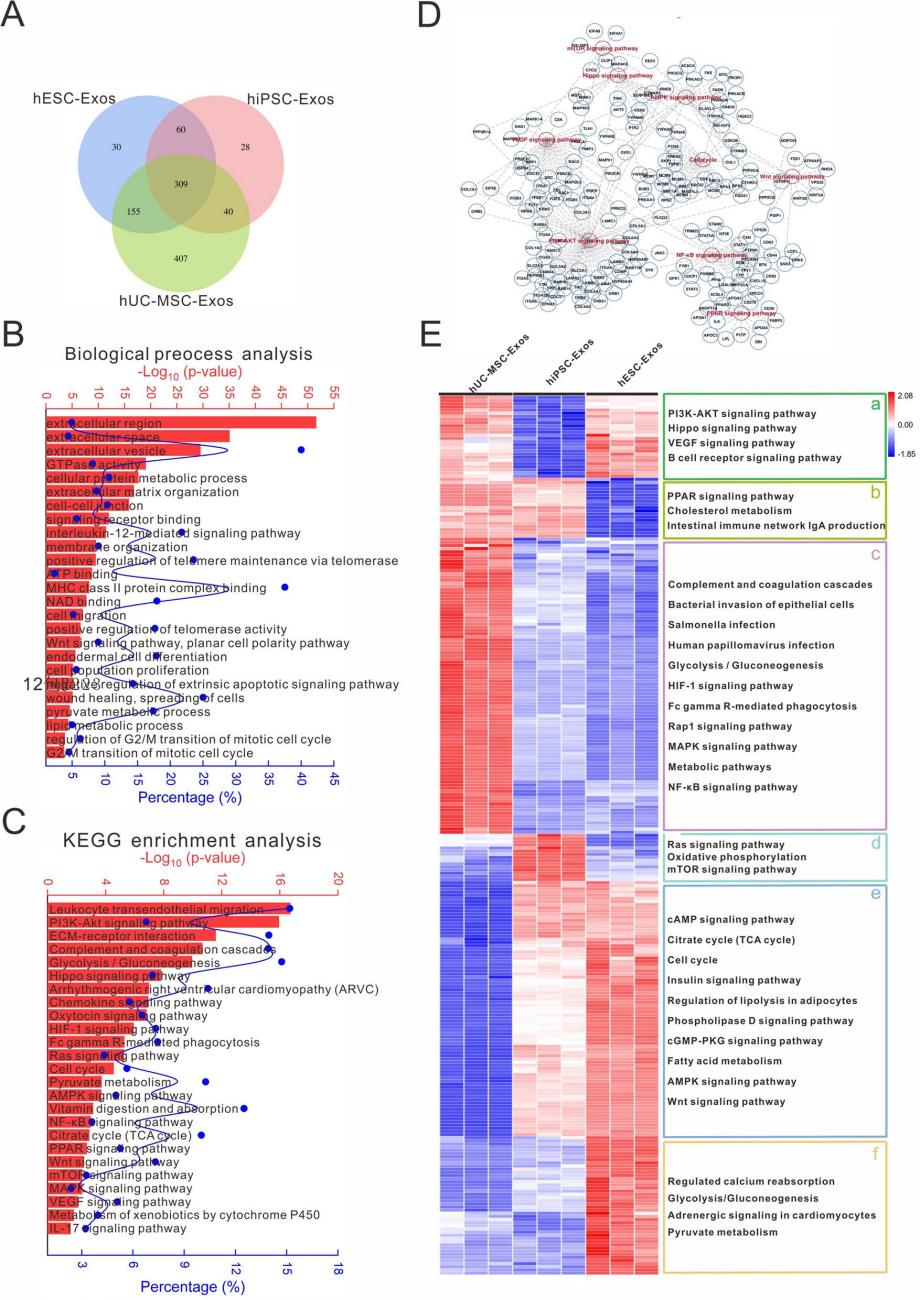
The overlapping proteins of three exosome samples were involved in complex biological regulation. **A** Venn diagram of the shared proteins in hESC-Exos, hiPSC-Exos, and hUC-MSC-Exos. **B** and **C** GO and KEGG analyses of the shared proteins among the three exosome types. The red bar represents the -Log_10_ (*P*-value) and the blue dot the proportion of candidate genes in the total pathway-related gene pool. **D** Crosstalk between the shared proteins in hESC-Exos, hiPSC-Exos, and hUC-MSC-Exos and regulated the signaling pathways they regulate. **E** Heatmap of the levels of shared proteins among the three exosome types. The significantly enriched pathways are shown on the right

Next, we investigated the shared proteomes of the three exosome types. In total, 309 protein-coding genes (Fig. 4A) were subjected to GO and KEGG analyses. The results indicated that shared protein-coding gene sets among the three exosome types were mainly involved in extracellular activities and biological processes including cellular protein metabolic processes, signaling receptor binding, ATP binding, NAD binding, wounding healing, and lipid metabolic processes (Fig. 4B). They also contributed to the construction of complex regulatory networks of signaling pathways such as PI3K-AKT, glycolysis/gluconeogenesis, Hippo, Oxytocin, HIF-1, cell cycle, and AMPK pathways (Fig. 4C). There were intricate regulatory relationships between these proteomic and canonical signaling pathways (Fig. 4D). The bubble plot evidences the different abundance of each protein cluster in canonical signaling pathway. Whereas hESC-Exos and hiPSC-Exos might have stronger regulatory abilities than hUC-MSC-Exos in terms of cell cycle and AMPK signaling pathways, hUC-MSC-Exos might have prominent regulatory effects on the VEGF and NF-κB signaling pathways (Additional file 1: Fig. S8).

The heatmap analysis further revealed differences in the expression of shared proteins (Fig. 4E). In Cluster a, proteins involved in the PI3K-AKT, Hippo, VEGF, and B-cell receptor signaling pathways were enriched in hUC-MSC-Exos and hESC-Exos. Proteins in Cluster b mainly participated in the regulation of PPAR signaling, cholesterol metabolism, and IgA production and were depleted in hESC-Exos. The hUC-MSC-Exos mainly contained Cluster c proteins, which regulated the complement response, microbial infection, HIF-1 signaling, MAPK signaling, metabolic pathways, and the NF-κB pathway. Proteins in Cluster d, which were enriched in hiPSC-Exos, participated in regulating Ras signaling, oxidative phosphorylation, and mTOR signaling. Proteins in Cluster e were more abundant in both hiPSC-Exos and hESC-Exos than in hUC-MSC-Exos, and they were involved in multiple metabolic processes, such as the citrate cycle, cell cycle, insulin signaling, fatty acid metabolism, and AMPK signaling. Proteins in Cluster f participated in regulating calcium reabsorption, glycolysis/gluconeogenesis, adrenergic signaling, and pyruvate metabolism and were depleted in both hUC-MSC-Exos and hiPSC-Exos. Proteins in the different clusters are listed in Additional file 4.

### miRNA profiles of the three exosome types

**Fig. 5:**
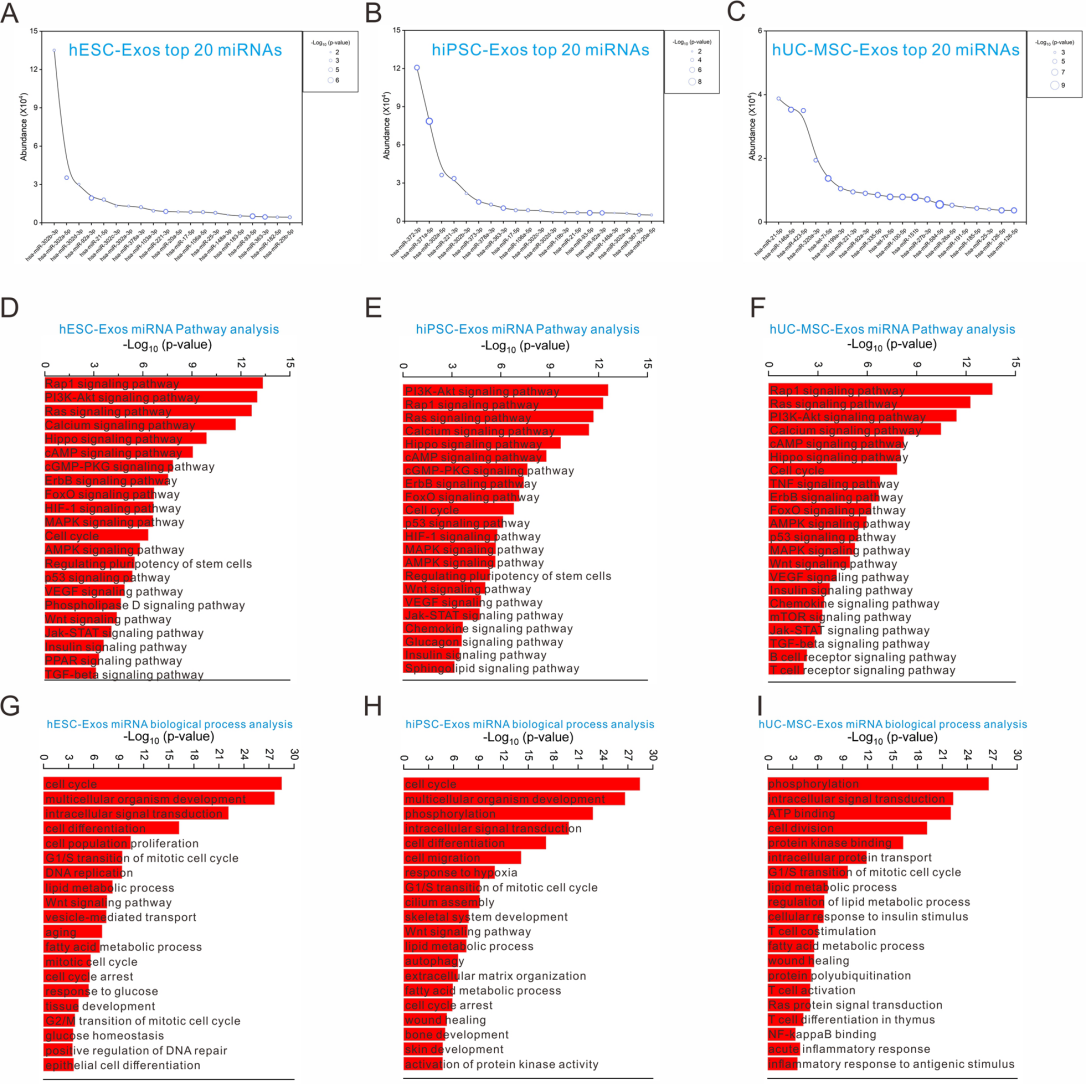
The top miRNAs derived from three exosome samples finely regulate complex signaling network. **A**–**C** Abundance of the top 20 miRNAs in hESC-Exos (**A**), hiPSC-Exos (**B**), and hUC-MSC-Exos (**C**). **D**–**F** Pathways regulated by the top miRNAs (read > 1000) in hESC-Exos (**D**), hiPSC-Exos (**E**), and hUC-MSC-Exos (**F**). **G**–**I** Biological processes regulated by the top miRNAs (read > 1000) in hESC-Exos (**G**), hiPSC-Exos (**H**), and hUC-MSC-Exos (**I**). The red bar represents the -Log_10_ (*P*-value)

To investigate the miRNA profiles of the three exosome types, total RNA was isolated from exosomes to couple the 5’ and 3’ ends for subsequent inverse transcription. The cDNA obtained was used for library construction and broad testing. RNA integrity number (RIN) was 2.7, 2.6, and 2.6 in hESC-Exos, hiPSC-Exos, and hUC-MSC-Exos, respectively (Additional file 1: Fig. S9A). Determination of RNA concentration revealed that hESC-Exos dominated the RNA load, followed by hiPSC-Exos and hUC-MSC-Exos (Additional file 1: Fig. S9B). Pearson’s correlation coefficient (Additional file 1: Fig. S9C) and PCA (Additional file 1: Fig. S9D) were used to evaluate the repeatability of miRNAs quantitatively. Heatmaps were used to represent the differentially expressed miRNAs in the three exosome types (Additional file 1: Fig. S9E), and the number of differentially expressed miRNAs at *P* < 0.05 or 0.01, was evaluated between each two exosome types (Additional file 1: Fig. S9F). Based on the sequencing results, the top 20 miRNAs in each of the three exosome types were identified (Fig. 5 A–C). In hESC-Exos, has-miR-302 dominated the read counts and comprised has-miR-302b-3p, has-miR-302a-5p, and has-miR-302d-3p (Fig. 5A). The three miRNAs with the highest read counts were has-miR-372-3p, has-miR-371a, and has-miR-302a in hiPSC-Exos and has-miR-21-5p (Fig. 5B), has-miR-146a-5p, and has-miR-320a-3p in hUC-MSC-Exos (Fig. 5C).

To investigate the miRNAs involved in biological processes, the top miRNAs were filtered at *P* < 0.05 and read count > 1000 for further target gene prediction. Enriched genes were then subjected to GO and KEGG analyses. The results indicated that hESC-Exos miRNAs significantly affected the following processes: Rap1, PI3K-AKT, calcium, Hippo, cAMP, ErbB, Foxo, cell cycle, AMPK signaling pathway, etc*.* (KEGG analysis) (Fig. 5D), and the cell cycle, multiple organism development, intracellular signal transduction, cell differentiation, aging, Wnt signaling, and fatty acid metabolism*.* (biological process) (Fig. 5G). Target gene enrichment of hiPSC-Exos miRNAs indicated that the following processes were significantly regulated: PI3K-AKT, Rap1, Ras, Calcium, Hippo, cAMP, and cGMP-PKG signaling pathway, etc*.* (KEGG analysis), and the cell cycle, multiple organism development, phosphorylation, cell differentiation, cell migration, and response to hypoxia*.* (biological process) (Fig. 5H). In the regulatory network of hUC-MSC-Exos miRNAs, the most plausible biological processes were: Rap1, Ras, PI3K-AKT, calcium, cAMP, Hippo, the cell cycle, JAK-STAT, and HIF-1 signaling pathway*.* (KEGG analysis) (Fig. 5F), and phosphorylation, intracellular signal transduction, ATP binding, cell division, lipid metabolism, T cell co-stimulation, wound healing, NF-κB binding, and inflammatory response*.* (biological process) (Fig. 5I). The network of the top five miRNAs and their regulatory pathways was also depicted (Additional file 1: Fig. S10).

### Unique and shared miRNAs of the three exosome types

**Fig. 6:**
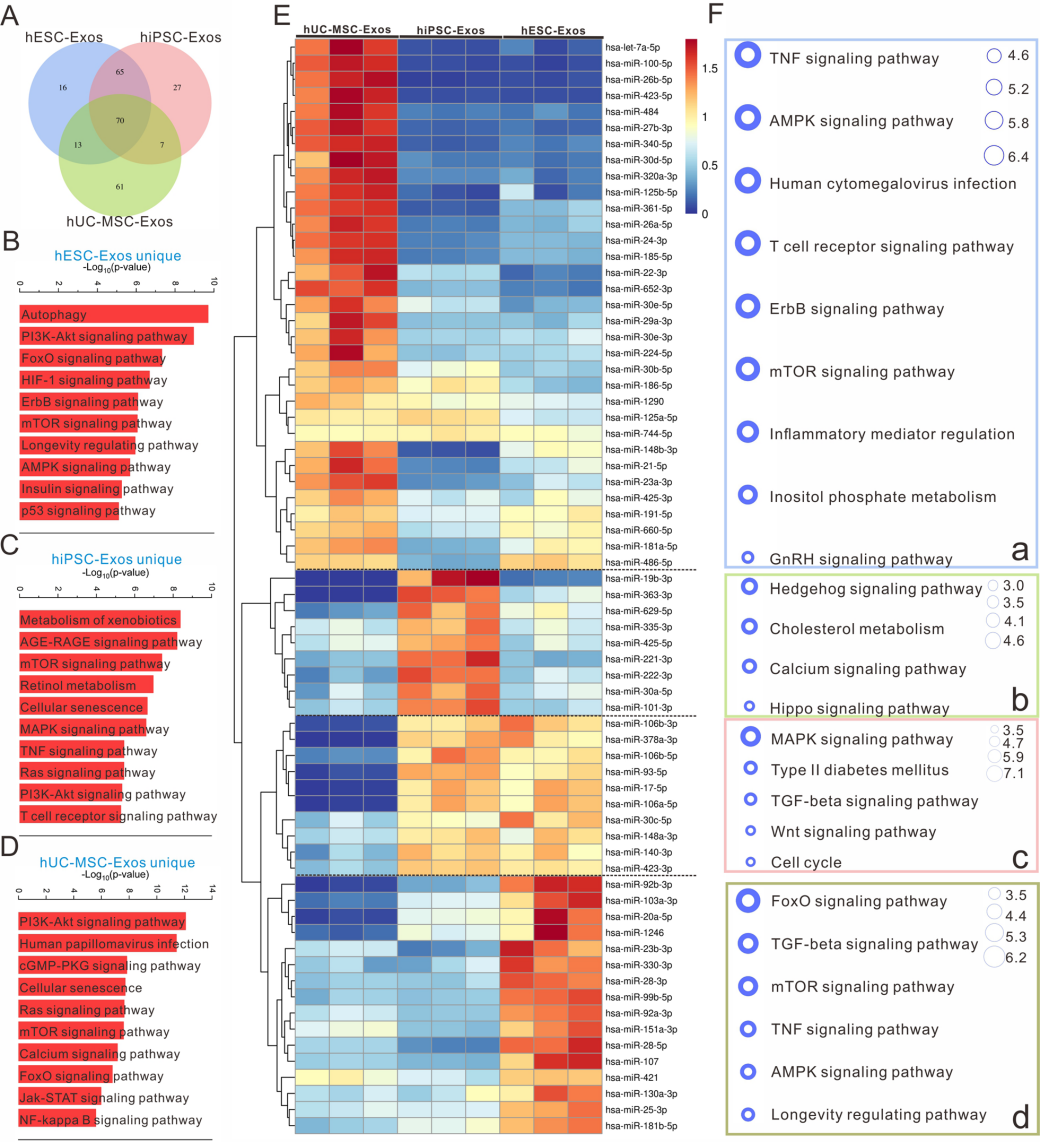
The signal regulation characteristics of specific miRNAs derived from three exosome samples. **A** Venn diagram of the miRNAs in hESC-Exos, hiPSC-Exos, and hUC-MSC-Exos. **B**–**D** Pathways regulated by the unique miRNAs in hESC-Exos (**B**), hiPSC-Exos (**C**), and hUC-MSC-Exos (**D**). The red bar represents -Log_10_ (*P*-value). **E** Heatmap of the shared miRNAs among the three exosome types. **F** Significantly enriched pathways regulated by the different miRNA clusters in (**E**). The bubble size represents -Log_10_ (*P*-value)

Next, we investigated the role of the unique miRNAs from the three exosome types in signal regulation. The Venn diagram revealed 16, 27, and 61 unique miRNAs in hESC-Exos, hiPSC-Exos, and hUC-MSC-Exos, respectively, and 70 shared miRNAs (Fig. 6A). The regulatory network of miRNA-protein interactions was evaluated by targeting the unique miRNAs. In hESC-Exos, the 16 unique miRNAs were found to regulate autophagy, PI3K-AKT, Foxo, HIF-1, ErbB, mTOR, longevity, AMPK pathway, etc*.* (Fig. 6B). For the 27 unique miRNAs in hiPSC-Exos, KEGG analysis revealed multiple significant ontologies, including metabolism of xenobiotics, AGE-RAGE signaling, mTOR signaling, retinol metabolism, cellular senescence, MAPK signaling, etc*.* (Fig. 6C). In hUC-MSC-Exos, the 61 unique miRNAs were found to participate in the regulation of PI3K-AKT signaling, human papillomavirus infection, cGMP-PKG signaling, cellular senescence, Ras signaling, mTOR signaling, JAK-STAT signaling, NF-κB signaling, etc*.* (Fig. 6D).

The miRNA components indirectly contribute greatly to multiple signaling pathways. To investigate the interaction between the unique miRNA clusters and canonical pathways, we performed a regulatory network analysis using the IPA database (https://www.ipaglobal.com/about/databases/). IPA results revealed that the top read count miRNAs (*P* < 0.05, abundance > 1000) in the regulatory network were mapped to multiple canonical signaling pathways. miRNAs and their corresponding pathways are listed in Additional file 5. Using hESC-Exos miRNAs as examples, multiple high-abundance miRNAs, including has-miR-95-3p, has-miR-30a-3p, has-miR-181-5p, has-miR-183-3p, and has-miR-301a-3p, were involved in AMPK, autophagy, ErbB, longevity regulation, and the FOXO signaling pathway, among others. Cytoscape was used to draw the network of unique miRNAs from the three exosome types and their regulated signaling pathways (Additional file 1: Fig. S11).

### miRNA profiles related to pluripotency regulation

**Fig. 7:**
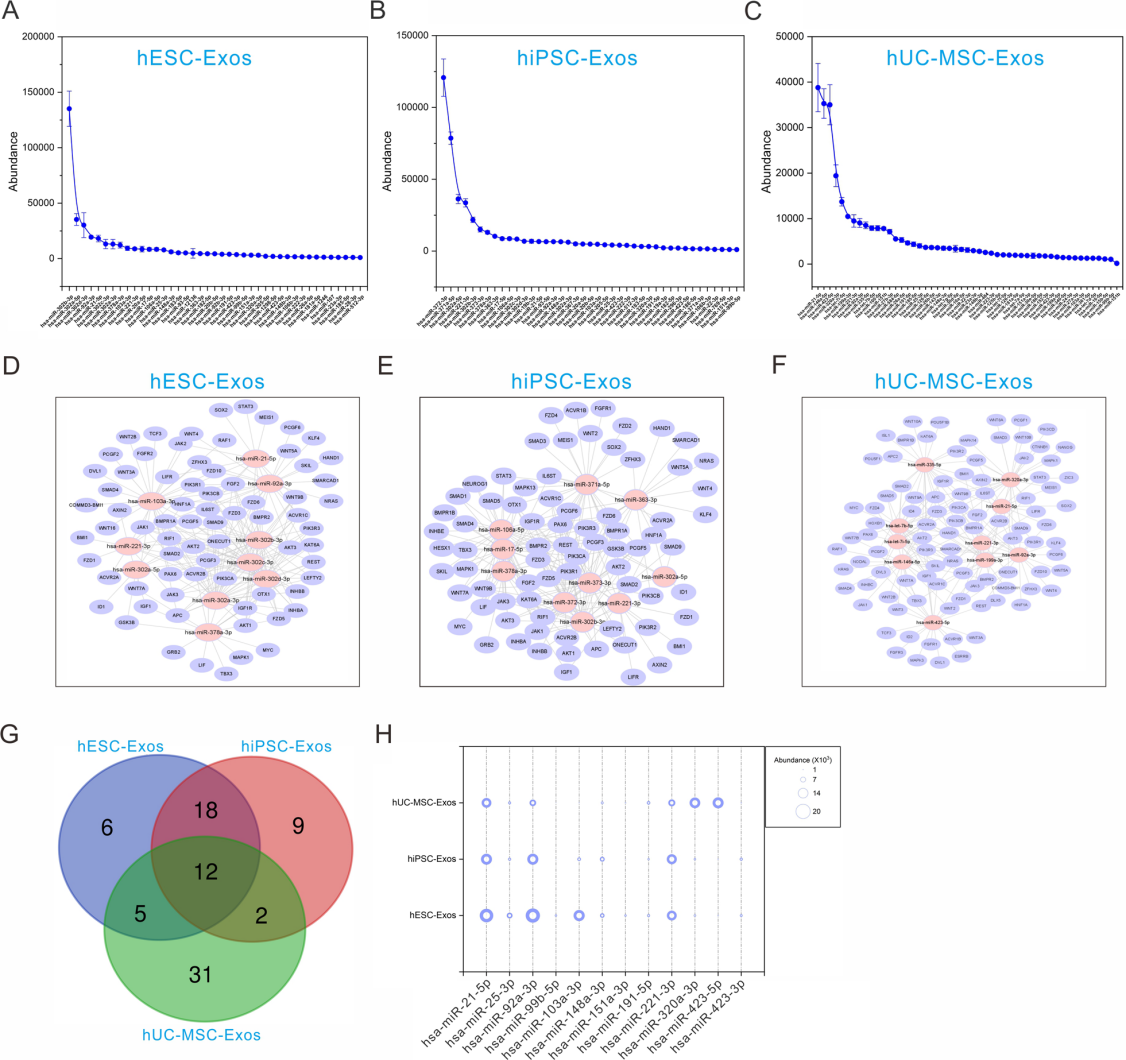
Three exosome samples had different miRNA profiles related to pluripotency regulation. **A**–**C** Abundance of the miRNAs related to pluripotency regulation. **D**–**F** Regulatory network of the top ten miRNAs and their target genes in pluripotency regulation. **G** Venn diagram of the miRNAs involved in pluripotency regulation and shared among the three exosome types. (**F**) Abundance of the miRNAs in (**G**). The bubble size represents abundance

To investigate the regulatory effect of exosome-derived miRNAs on the pluripotency of stem cells, we predicted the target genes and screened the miRNAs involved in regulating the above process. Pluripotency-related miRNAs (abundance > 1000) in the three exosome types are listed (Fig. 7A–C). The top three miRNAs in the three exosome types were has-miR-302b-3p, has-miR-302a-5p, and has-miR-302d-3p (hESC-Exos), has-miR-372-3p, has-miR-371a-5p, and has-miR-221-3p (hiPSC-Exos), and has-miR-21-5p, has-miR-146a-5p, and has-miR-320a-3p (hUC-MSC-Exos). Furthermore, we described the regulatory network of the top ten miRNAs from exosomes and their target genes involved in pluripotency regulation (Fig. 7D–F). Venn diagram analysis revealed 12 overlapping miRNAs, which indicated that they might be crucial miRNA sets in regulating the pluripotency of stem cells (Fig. 7G–H).

### Specific proteins and miRNAs in the three exosome types

**Fig. 8:**
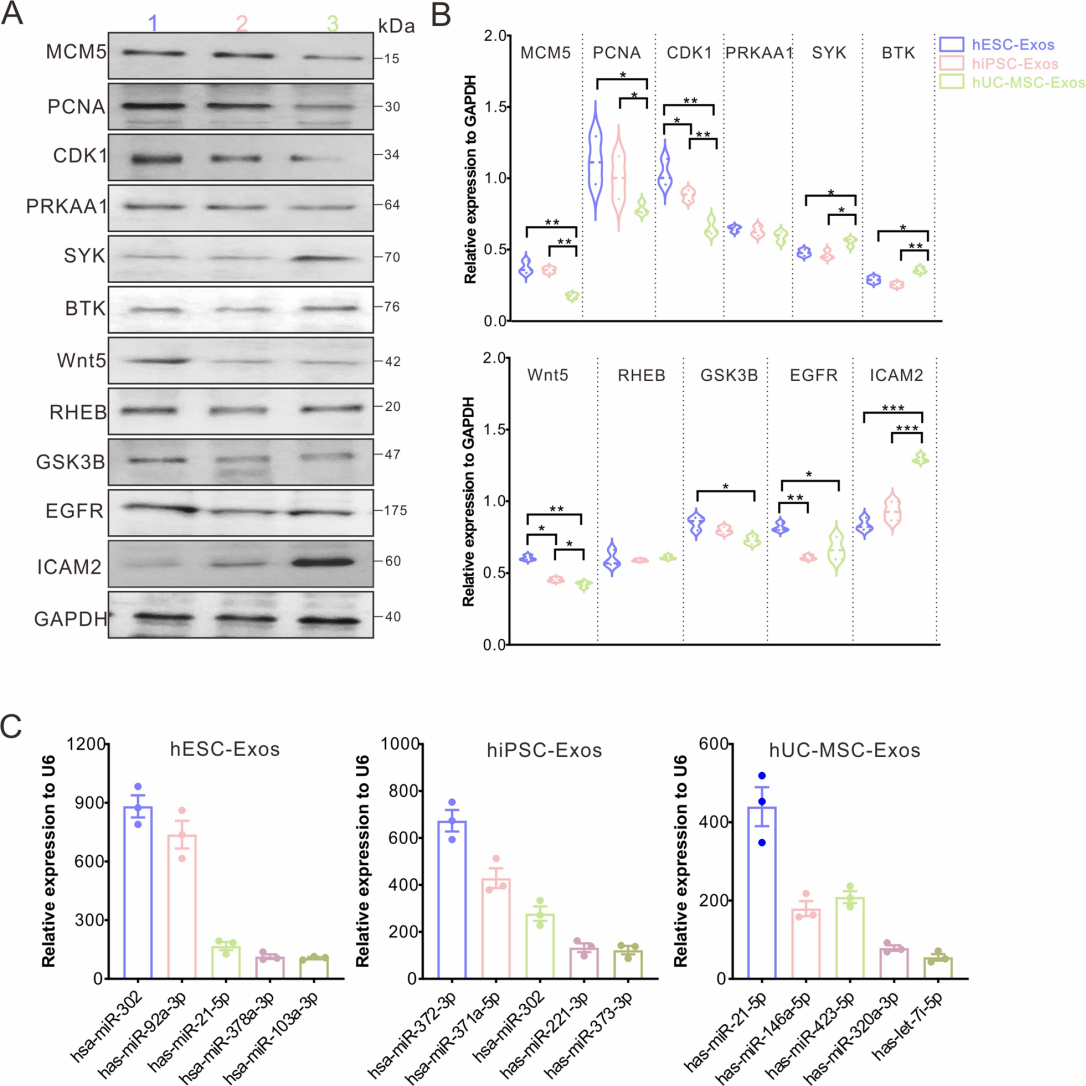
Detection of specific proteins and miRNAs in the three exosome types. **A** Representative western blot graphs regarding the detection of representative proteins in hESC-Exos (1), hiPSC-Exos (2), and hUC-MSC-Exos (3). GAPDH was set as the internal reference. Three independent replicates were performed. **B** Quantification of protein levels in (**A**). All statistical data are presented as means ± standard deviation of two-tailed unpaired Student’s *t*-tests. ^*^P < 0.05, ^**^P < 0.01, and ^***^P < 0.001. **C** RT-qPCR detection of the expression of the top five miRNAs in the three exosome types. Three independent replicates were performed

To further verify the actual expression of specific proteins in the three exosomes, we performed western blotting to detect candidate proteins in different pathways (Fig. 8A and B). For the cell cycle, three crucial regulatory factors, MCM5, PCNA1, and CDK1, were more highly expressed in hESC-Exos and hiPSC-Exos than in hUC-MSC-Exos. PRKAA1, belonging to the ser/thr protein kinase family, is a cellular energy, conserved factor in all eukaryotic cells and showed a similar load in the three exosomes. SYK is widely expressed in hematopoietic cells and is involved in the coupling of activated immunoreceptors to downstream signaling events. BTK plays a crucial role in B cell development and immunoregulation. The protein loads of SYK and BTK were elevated in hUC-MSC-Exos compared to those in hESC-Exos or hiPSC-Exos. Wnt5 is a member of the Wnt gene family, which has been implicated in developmental processes, including the regulation of cell fate and patterning during embryogenesis. hESC-Exos were the most enriched in Wnt5, followed by hiPSC-Exos and hUC-Exos. RHEB is vital in regulating growth and cell cycle progression given its role in the mTOR/S6K signaling pathway. Western blotting indicated that the three exosome types carried a similar RHEB load. EGFR is a cell surface protein that binds the epidermal growth factor, thus inducing receptor dimerization and tyrosine autophosphorylation, leading to cell proliferation. Both hESC-Exos and hUC-MSC-Exos had higher EGFR levels than hiPSC-Exos did. ICAM2 is a member of the intercellular adhesion molecule (ICAM) family and mediates adhesive interactions important for antigen-specific immune response, NK cell-mediated clearance, lymphocyte recirculation, and other cellular interactions important for immune response and surveillance. Among the three exosomes, hUC-MSC-Exos had the highest ICAM2 level. In addition, the top five miRNAs in the three exosome types were evaluated by RT-qPCR, and their expression was consistent with the RNA sequencing results (Fig. 8C).

## Discussion

EVs are rich in various biologically active substances and are mainly composed of proteins, nucleotides, and lipids [3, 17]. In recent decades the research community has ushered in the golden age of analytical techniques for protein, nucleotide, and lipid omics. Among these techniques, mass spectrometry [14, 20] and high-throughput sequencing [6, 54] enable the large-scale screening and identification of EV components. Two databases, Vesiclepedia (https://microvesicles.org) [28] and EVpedia (https://evpedia.info) [29], are continuously updated and provide a summary of the components of mammalian and non-mammalian EVs, respectively. As EVs, exosomes differ from MVs in terms of their biological origin and physical dimensions [46]. With continuous advances in the field of EVs, new methods are constantly being optimized to facilitate the isolation and purification of exosomes, meeting the rigor need for comparative analysis. To replenish the exosome databases and expand the scope of their clinical applications, in this study we describe the component framework of exosomes from hESCs and hiPSCs, which has not yet been reported. We also compared their composition and biological functions with those of hUC-MSC-derived exosomes. To this end, we arrested exosomes from EVs at recognized sizes using ultracentrifugation combined with filtration, excluding MVs and apoptotic bodies, which facilitated the refinement of exosome components. During the logarithmic growth phase of the cells, the collected CMs were ready for exosome preparation. The particle concentration of hESC-Exos was found to be significantly higher than that of hUC-MSC-Exos, but similar to that of hiPSC-Exos. Similar results were obtained when comparing the protein concentrations. In contrast, total RNAs exhibited a significant decrease in gradients from hESC-Exos and hiPSC-Exos to hUC-MSC-Exos. These results indicate that hESCs (H9) may play a central role in the replication activity and have a stronger ability to secrete exosomes among the three stem cells.

In previous studies, researchers have focused on the accessibility of exosomes [1, 11, 58] while being confined to their research field. As a result, less attention has been paid to difference analysis between exosomes derived from different sources. Although some EV proteomics of the three stem cells has been performed, researchers have not yet refined the dimension of exosomes [19, 32, 59], and the assay method is generally based on a single mass spectrometry technique. In the present study, both TMT and LFQ methods were used to measure and quantify the protein components of the isolated exosomes. Bioinformatics analysis revealed that highly loaded proteins could coordinate the processes of development, damage repair, and metabolism via intervening Wnt, AMPK, VEGF, and cell cycle signaling pathways, which was consistent with a previous report [19]. Similarly, hiPSC-Exos could also participate in the above biological processes by affecting the collateral signals. However, the EV proteomics of hiPSCs induced from HFFs showed a different gene ontology overview, focusing more on the processes of DNA replication and RNA catabolism [45]. Therefore, we deduced that exosomes secreted from hiPSCs derived from different tissues may have different protein profiles. In terms of hUC-MSCs-Exos, its components blended into many immunomodulatory activities by regulating complement system, microbial infection, NF-κB signaling, and so on, and affected multiple metabolic signals, such as cholesterol metabolism, phospholipase D metabolism, and purine metabolism, providing results similar to those of previous reports [1, 59].

In the present study, we compared the proteomes of three types of exosomes pairwise and further analyzed their exclusive proteins regarding functional pathways and regulatory networks. The hiPSCs are a type of pluripotent stem cells that can be generated by reprogramming somatic cells to mimic the pluripotency of hESCs [48], which shows that these cells are similar to a certain extent. Although their exosome proteins were highly overlapping, those of hESC-Exos were enriched in developmental regulation and the cell cycle functions, indicating that hESC-Exos might have a stronger ability to regulate pluripotency than hiPSC-Exos in our study. The pluripotency of hUC-MSCs is inferior to that of hiPSCs and hESCs, as reflected by their notably different protein profiles. The proteome of hUC-MSC-Exos differed from that of hESC-Exos and hiPSC-Exos in immunoregulation, being enriched in proteins regulating the activities of natural killer cells and the complement system. Furthermore, bioinformatics of the proteins shared by the three exosome types revealed that the upregulation of hESC-Exos or hiPSC-Exos proteins focused more on metabolism, development, and cell proliferation functions by interfering with classical signaling pathways such as the AMPK, Wnt, mTOR, and the cell cycle. In comparison, the upregulated proteins of hUC-MSC-Exos were not only enriched in immune-related signaling, including the complement system, microbial infection, NF-κB signaling, and B cell receptor signaling, but also in metabolic processes such as PPAR signaling, cholesterol metabolism, and MAPK signaling.

Exosome cargos are of unique tissue and cellular origins and contain miRNAs [16]. The present study also showed that exosomes isolated from CMs produced in vitro by hESCs, hiPSCs, and hUC-MSCs contained distinctive and specific miRNA signatures. We found that each exosomal miRNAs had a unique landscape. miRNA expression and interaction with the 3’ or 5’ UTR of their target genes are involved in complex physiological and pathophysiological activities[18]. The top-loaded miRNAs of the three exosome types regulate a series of biological processes, including the cell cycle and Hippo, Wnt, AMPK, and TGF-β signaling. However, the miRNA profiles of hUC-MSC-Exos had a stronger immunomodulatory ability than that of hESC-Exos or hiPSC-Exos regarding the behaviors of B and T cells, as well as TNF, JAK-STAT, and NF-κB signaling. In particular, most unique miRNA profiles were linked to the regulation of autophagy, longevity, and PI3K-Akt, mTOR, AMPK, and p53 signaling, indicating that these miRNA clusters may modulate aging, aging-related diseases, tissue repair after injury, and metabolism. The unique miRNAs of hiPSC-Exos regulate mTOR signaling, cellular senescence, retinol metabolism, and TNF signaling, which also contributes to senescence and metabolism regulation. In addition to mTOR signaling and cell senescence, the unique miRNAs of hUC-MSC-Exos regulate Foxo, Jak-STAT, and NF-κB signaling, which may contribute to remodeling the metabolic and immune microenvironment. The analysis of shared miRNAs among the three exosome types further supported these differences in signaling regulation. Overall, the miRNA clusters in hESC-Exos or hiPSC-Exos coordinated the occurrence of multiple events, such as development, cell cycle, and cell differentiation. The miRNA set of hiPSC-Exos seem to play less important role in these functions than that of hESC-Exos but a more important role than that of hUC-MSC-Exos. As for hUC-MSC-Exos, the miRNA profiles found indicate their superior ability in regulating the immune environment, particularly in wound and infection healing.

In developmental biology, exosomes derived from pluripotent stem cells promote the maintenance of the pluripotent state. Therefore, miRNAs entering the cell microenvironment contribute significantly to the maintenance of stemness [4, 32]. The three exosome types in the present study contained different miRNA clusters that regulate pluripotent signaling. Nevertheless, the 12 miRNAs shared among the three exosome types may be crucial for pluripotency regulation, including miR-21-5p, miR-92a-3p, and miR-221-3p. This hypothesis remains to be tested, and more stem cell types need to be investigated. In addition, the overlapping miRNAs between hESC-Exos and hiPSC-Exos may be involved in cell differentiation and reprogramming, as indicated by the results of more detailed analyses. For example, the miR-302 family was highly enriched in and exclusive to hESC-Exos and hiPSC-Exos and contributed greatly to influencing stem cell behavior by modulating reprogramming [33, 50]. This point indirectly matches previous research regarding the uniqueness of miR-302 in human and mouse ESCs [33, 50]. Expression analysis of miRNA clusters that were highly expressed in ESCs during the initial phase of reprogramming revealed the induction of miR-17 [41] and miR-106a/106b [37]. Overexpression of miR-93 promoted an increase in the colony number of iPSCs [35].

The signaling network established by exosome cargos drives intracellular events, further intervening in a series of pathological and physiological processes [3]. The exosomes of hESCs contain numerous loaded proteins and miRNAs predicted to regulate the landscape of development, metabolism, and anti-aging via blending into the AMPK, mTOR, and Wnt signaling pathways and regulating autophagy, longevity, and the cell cycle. Multiple studies have highlighted the functions of hESC-EVs in rejuvenating the aging hippocampus [25, 26], bone marrow [19], and endothelial cells [10], as well as alleviating recurring osteoarthritis by delivering specific proteins or miRNAs [58]. Our findings also support previous research, in which ESC-derived EVs were found to have positive implications in restoring impaired cardiovascular function [5]. ESC-derived EVs enabled maintaining the stemness of ESCs, thus being capable of reprogramming [4], which is in line with our results for hESC-Exos cargos. In our study, hiPSCs reprogrammed from umbilical cord cells had biological functions similar to those of hESC-Exos; however, no reports have specifically supported this theory. In comparison, hUC-MSC-Exos have received more attention, and various preclinical and clinical trials have clarified their therapeutic effect on multiple diseases [51]. Although hUC-MSC-Exos contribute to tissue regeneration and tissue remodeling, these abilities are inferior to that of hESC-Exos and hiPSC-Exos. Our analysis revealed that hUC-MSC-Exos exhibited excellent immune regulation ability. These findings provide a basis for further research on inflammation-related diseases such as COVID-19 [2].

## Conclusions

Overall, hESC-Exos is outstanding in regulating development, metabolism, and anti-aging, hiPSC-Exos has similar biological function, but inferior to hESC-Exos. In comparison, hUC-MSC-Exos contribute more to immune regulation. Our analysis broadens the application scope of hESC-Exos, hiPSCs, and hUC-MSC-Exos and highlights their respective advantages in the intervention of disease-related signaling. To the best of our knowledge, this study is the first to report a systematic and comprehensive analysis of exosome proteomics and miRNA profiles of hESCs, hiPSCs, and hUC-MSCs. Our study further enriches the current EV databases, facilitating the mining of more valuable data for the identification of appropriate acellular therapies in clinical settings. These exosomes also cater for the drug development as an alternative delivery system to replace virus delivery system like adenovirus [7]. Although current predictions lack substantial validation, these findings could reveal further individual or joint applications of the three exosomes in preclinical or clinical research. Moreover, future research could also be conducted via the integrated differential analysis of exosome cargos with EVs, as well as of the components of the cells themselves.

## Supplementary Information

**Additional file 1.** Supplementary materials including methods and results.

**Additional file 2.** The measurement results of TMT assay.

**Additional file 3.** The measurement results of LFQ assay.

**Additional file 4.** The protein clusters in Figure 4E.

**Additional file 5.** The top-loaded miRNAs derived from three types of exosomes and their regulated signalling pathways.

## Data Availability

All datasets used and/or analyzed during the current study are available from the corresponding author on reasonable request. All authors have confirmed that a citation for available data in references section was included.

## Abbreviations

MSC: Mesenchymal stem cell
hESCs: Human embryonic stem cells
hiPSCs: Human-induced pluripotent stem cells
hUC-MSCs: Human umbilical cord mesenchymal stem cells
TMT: Tandem mass tag
LFQ: Label-free relative peptide quantification
hESC-Exos: Human embryonic stem cell-derived exosomes
hiPSC-Exos: Human embryonic stem cell-derived exosomes
hUC-MSC-Exos: Human umbilical cord mesenchymal stem cell-derived exosomes
EVs: Extracellular vesicles
MVs: Microvesicles
Alix: Apoptosis-linked gene 2-interactin protein X
TSG101: Tumor susceptibility gene 101
HFFs: Human foreskin fibroblasts
CAMs: Cell adhesion molecules
TEM: Transmission electron microscopy
PDI: Polydispersity
TEAB: Tetraethylammonium bromide
DEPs: Differentially expressed proteins
FDR: False discovery rates
