# Supplementary material for: Systemic proteomics and miRNA profile analysis of exosomes derived from human pluripotent stem cells
Source: Stem Cell Res Ther. 2022 Sep 5;13:449. doi: 10.1186/s13287-022-03142-1 (PMC9444124; doi:10.1186/s13287-022-03142-1)
Supplement: Supplementary file 1 — Additional file 1. Supplementary materials including methods and results. [file 13287_2022_3142_MOESM1_ESM.docx]

**Supplementary materials for** **Systemic proteomics and miRNA profile analysis of exosomes derived from human pluripotent stem cells**

**Methods**

**Culture and identification of three different stem cells**

Umbilical cords were obtained from newborns born in hospitals. Prior to cord collection, the parents signed an informed consent form, which was approved by the ethics committee of the hospital. The umbilical cord matrix was cut into 2.0-mm^2^ tissue blocks. Collagenase II was added to a final concentration of 0.05%, and the blocks were digested at 37°C with agitation for 0.5 h. PBS was added, and the mixture was filtered and centrifuged. The pellet was then resuspended in serum-free ncMission hMSC medium (cat. no. RP02010; Nuwacell. Ltd, China) and seeded in a T-25 cm culture flask at 1 × 10^6^ cells/cm^2^. The 3^rd^ generation cells were subjected to adipogenic and osteogenic induction for 14 days according to the protocols of MesenCult^TM^ Adipogenic Differentiation Kit (cat. no. 05507; STEMCELL) and osteogenic stimulatory kit (cat. no. 05504; STEMCELL), respectively. Flow cytometry was used to assay the surface markers of the candidate cells. They were incubated with antibodies against CD90, CD73, CD105, CD34, CD14, CD19, CD45, and HLA-DR (BD Bioscience) for 20 min at 4°C and washed on Lyse Wash Assistant (LWA) (BD Bioscience). As a result, 20,000 events were acquired on a FACSAria IIIu (BD Bioscience), the data of which were analyzed using Flow Jo software.

The hESC line H9 and hiPSCs (cat. no. RC01001-B) were kindly provided by the Stem Cell Bank, Chinese Academy of Sciences, and NuwaCell. Ltd. China, respectively. The cells were expanded in serum- and feeder-free medium ncTarget (cat. no. RP01020; Nuwacell. Ltd, China) and planted on vitronectin (VTN) (cat. no. RP01002; Nuwacell. Ltd, China) at 37°C in a humidified incubator with 5% CO_2_. The culture medium was changed daily. When the cell confluence reached approximately 85%, the cells were digested using dissociation buffer (cat. no. RP01007; Nuwacell. Ltd, China) for 8 min and passaged as clumps in ncTarget medium on VTN coated plates. The cells were identified as previously described[3].

**Western blot**

# Western blotting was performed to detect the surface markers of isolated exosomes according to a previous study. The samples were lysed with RIPA solution containing 1 mM PMSF and protease inhibitor cocktail (cat. no. 5871; Cell Signaling Technology) on ice for 10 min. The protein concentration of the lysate was determined using the Bradford assay (Sigma, USA). The mixture of the prepared protein and 4× loading buffer (cat. no. P1015; Solarbio, China) was boiled for 10 min. The protein samples were homogenized in concentration, separated by SDS-PAGE, transferred onto nitrocellulose membranes (cat. no. LC2000; Millipore, USA), and incubated with the primary antibodies listed in Table S1. The membranes were then incubated with the appropriate HRP-conjugated secondary antibodies (1:10,000) (ZB-2301; ZSGB-BIO, China), and the signals were detected using the SuperLumina ECL HRP Substrate Kit (cat. no. k22030; Abbkine, USA).

**Quantitative real-time PCR**

The total RNA was extracted by Trizol (cat. no. RN0102, Aidlab Biotecnologies, China)) the method as previous description. And reverse transcribed to cDNA using miRNA First Strand cDNA Synthesis kit (cat. no. MR201, Vazyme, China) according to the manufacturer’s protocol. A quantitative real-time polymerase chain reaction (qPCR) assay was performed using the SYBR Premix Ex Taq™ II Kit (cat. no. RR820DS, TaKaRa) and the expression levels of RNA and microRNA were normalized to the level of U6. The primers used in the qRCR assay were listed in Table S 2.

**Results**

**Identification of hESCs, hiPSCs, and hUC-MSCs**

The identification of hESCs and hiPSCs was performed as previously described[3]. Their colonies positively expressed multiple pluripotency-related markers, such as OCT4, NANOG, revealed by flow cytometry. Moreover, western blot further detected the expression of marker proteins of hESCs and hiPSCs including OCT4, NANOG, and Sox2 (Fig. S 1 A and B). We then investigated the properties of hUC-MSCs as previously reported according to the identification criteria of hUC-MSC promulgated by the international society for cell therapy (ISCT)[1,2]. They exhibited typical adherent growth with a spindle shape. Flow cytometry revealed that they were also positive for CD73, CD90, CD44, and CD105, and negative for CD34, CD11b, CD45, and HLA-DR (Fig. S1).

**
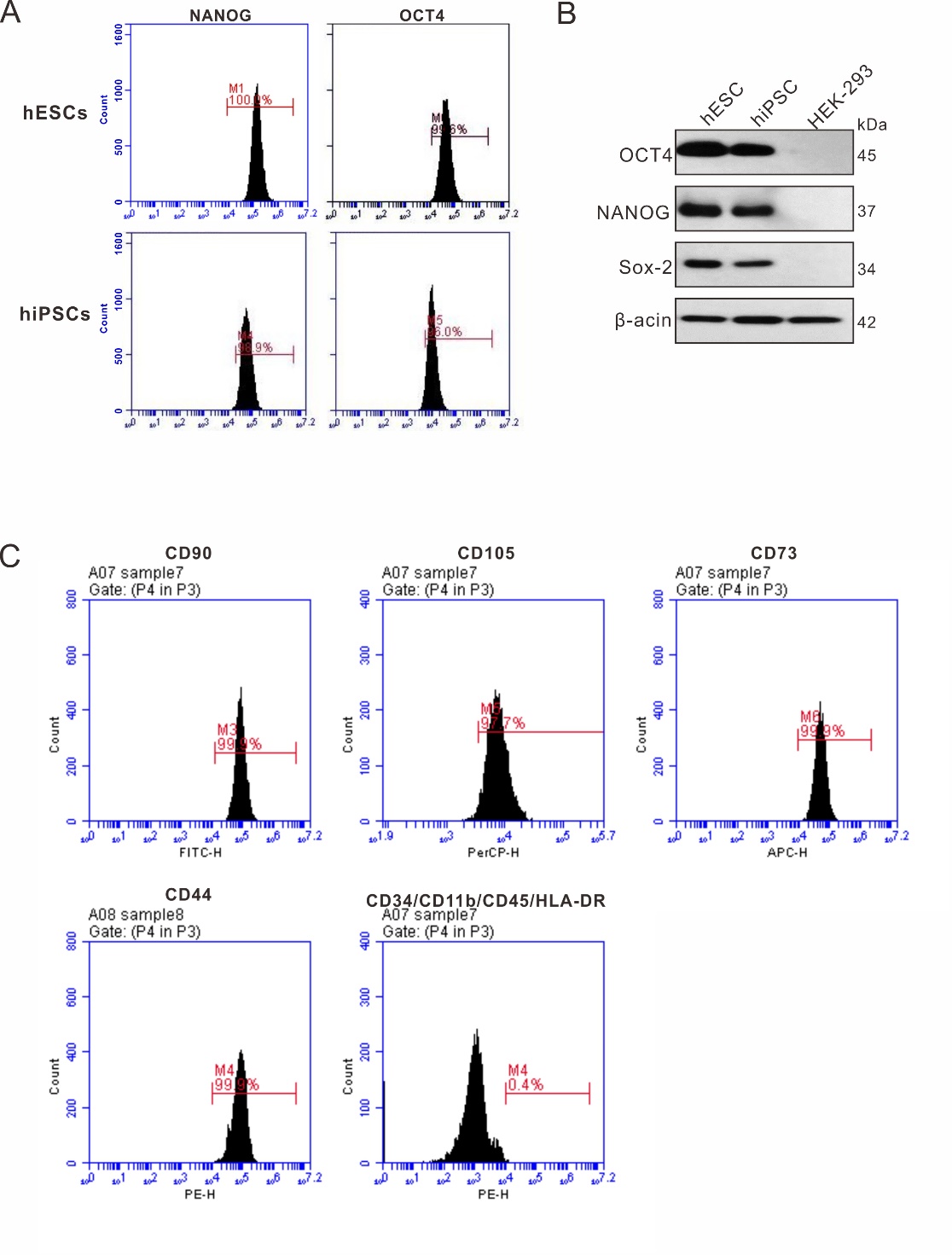
Fig. S1 Identification of hESCs, hiPSCs, and hUC-MSCs.** (A) The representative flow cytometry result for detecting the expression of marker genes (NANOG and OCT4) in hESCs and hiPSCs. (B) Western blot detected the expression of pluripotency-related genes, OCT4, NANOG, and Sox-2. β-actin was treated as an internal reference. (C) The representative flow cytometry analysis of cell-surface markers in the hUC-MSCs. All hUC-MSCs expressed positive cell markers including CD73, CD90, CD44and CD105, but negative for CD34, CD11b, CD45, and HLA-DR.


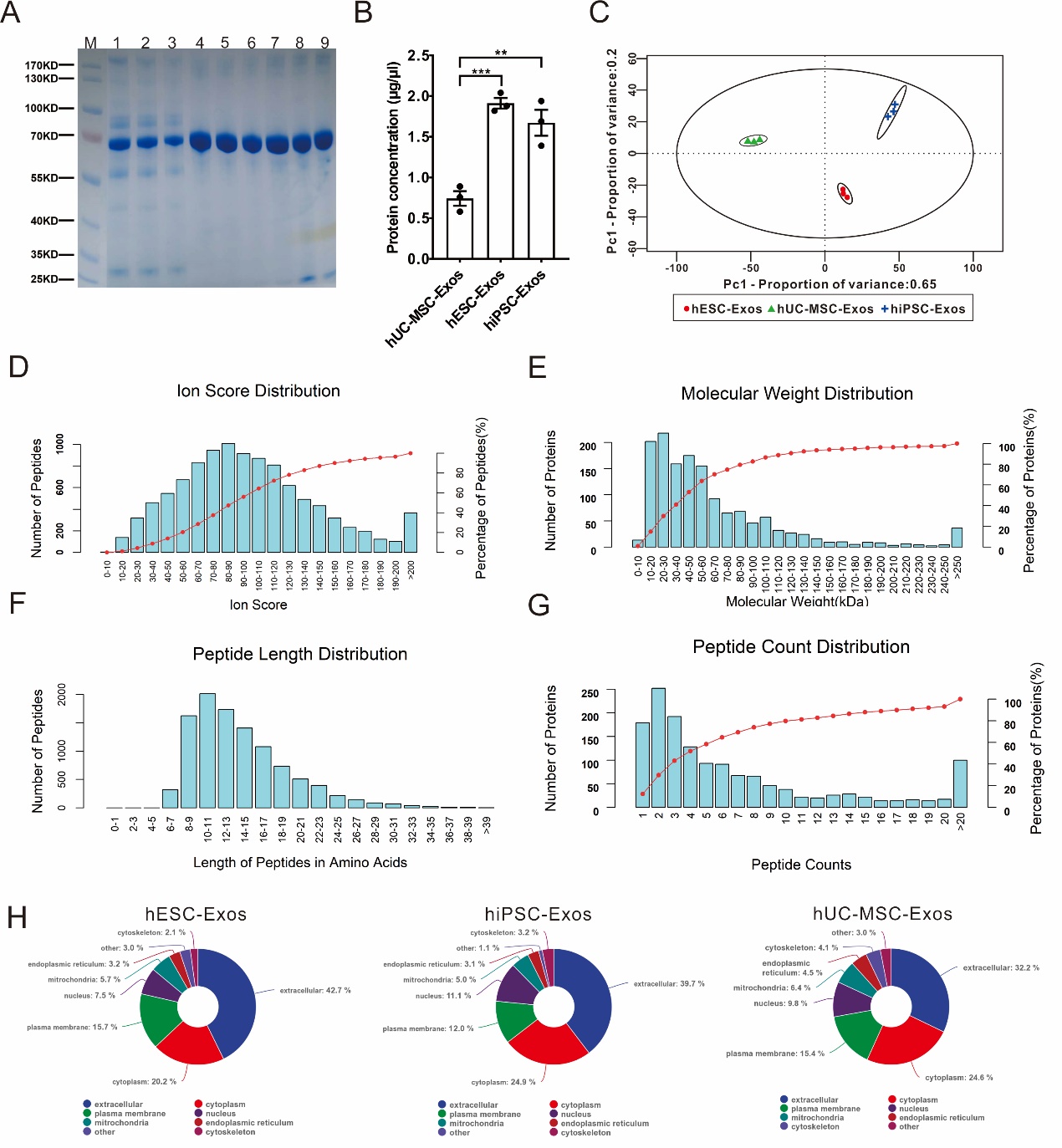
**Fig. S2 Quality inspection of protein components.** (A) Detection of protein distribution in three exosomes by Coomassie brilliant blue staining. 1-3, 4-6, and 7-9 represent hUC-MSC-Exos, hESC-Exos, and hiPSC-Exos, respectively. (B) The quantification of protein concentration in (A) (n = 3 samples per group). All statistical data are presented as means ± s and performed two-tailed unpaired Student’s *t*-test. ^*^*P*＜0.05, ^**^*P*＜0.01, and ^***^*P*＜0.001. (C) PCA map of proteins in three exosomes. (D-G) Quality inspection of peptide fragments digested via enzymatic hydrolysis in terms of Ion score distribution (D), Molecular weight distribution (E), Peptide length distribution (F), and Peptide count distribution(G). (H) The cellular localization of proteins derived from hESC-Exos, hiPSC-Exos, and hUC-MSC-Exos.


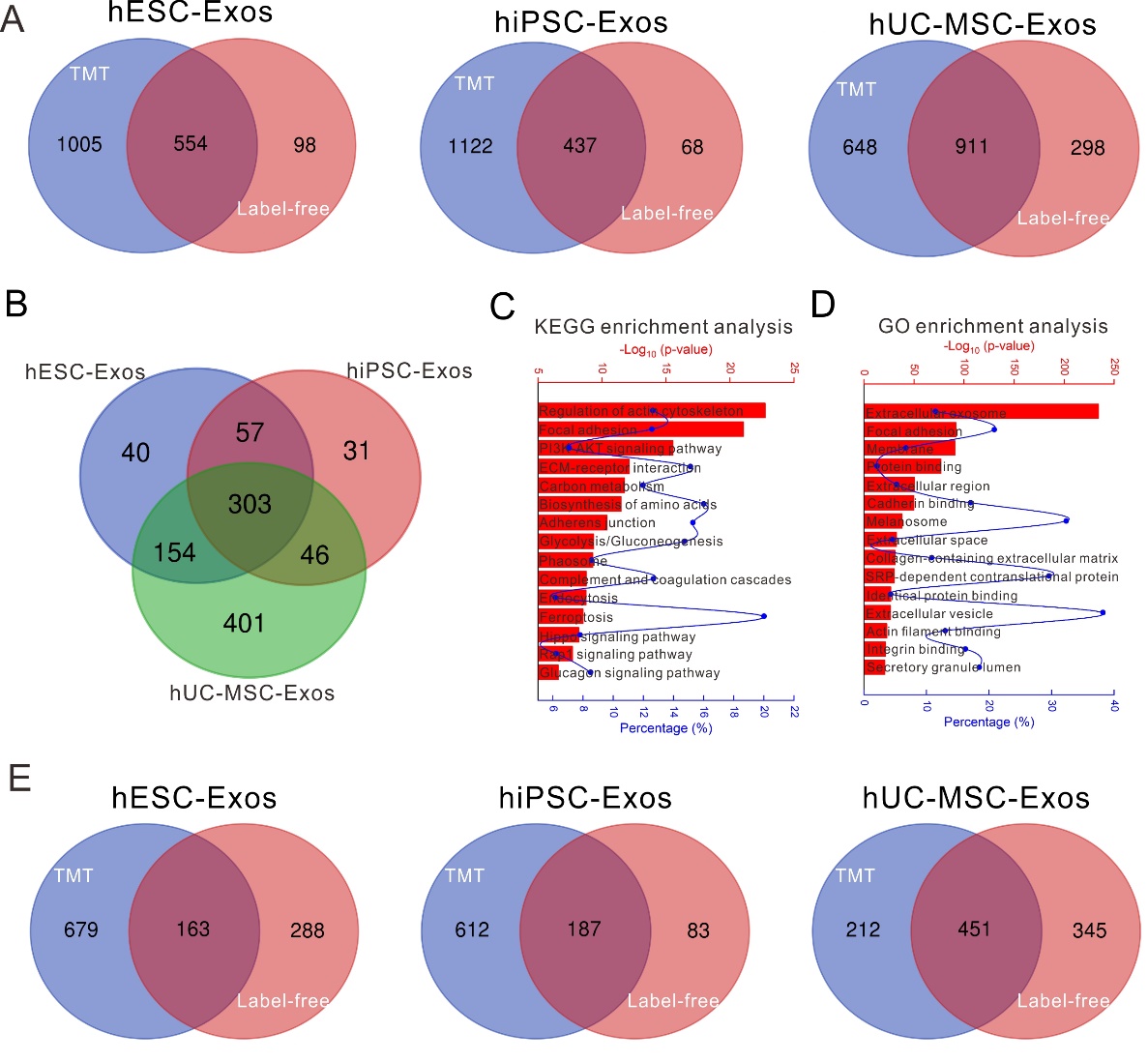
**Fig. S3 Data screening of protein components.** (A) Venn diagram of actual protein components in three exosomes. The actual protein components were screened under the condition of mascot score > 60 and abundance > 100 (TMT assay) and abundance of at least two repetitions > 0 (Label-free assay). (B) Venn diagram of selected proteins in (A). (C-D) KEGG and GO analysis of shared proteins in (B) according to the KOBAS algorithm. The red bar and blue dot represent the -Log_10_(*P*-value) and the proportion of candidate genes in the total pathway-related gene pool, respectively. (E) Venn diagram of top-loaded proteins in three exosomes. The top-expressed proteins were screened under the condition of high FDR confidence (TMT assay) and abundance of each repetition > 0 (Label-free assay).


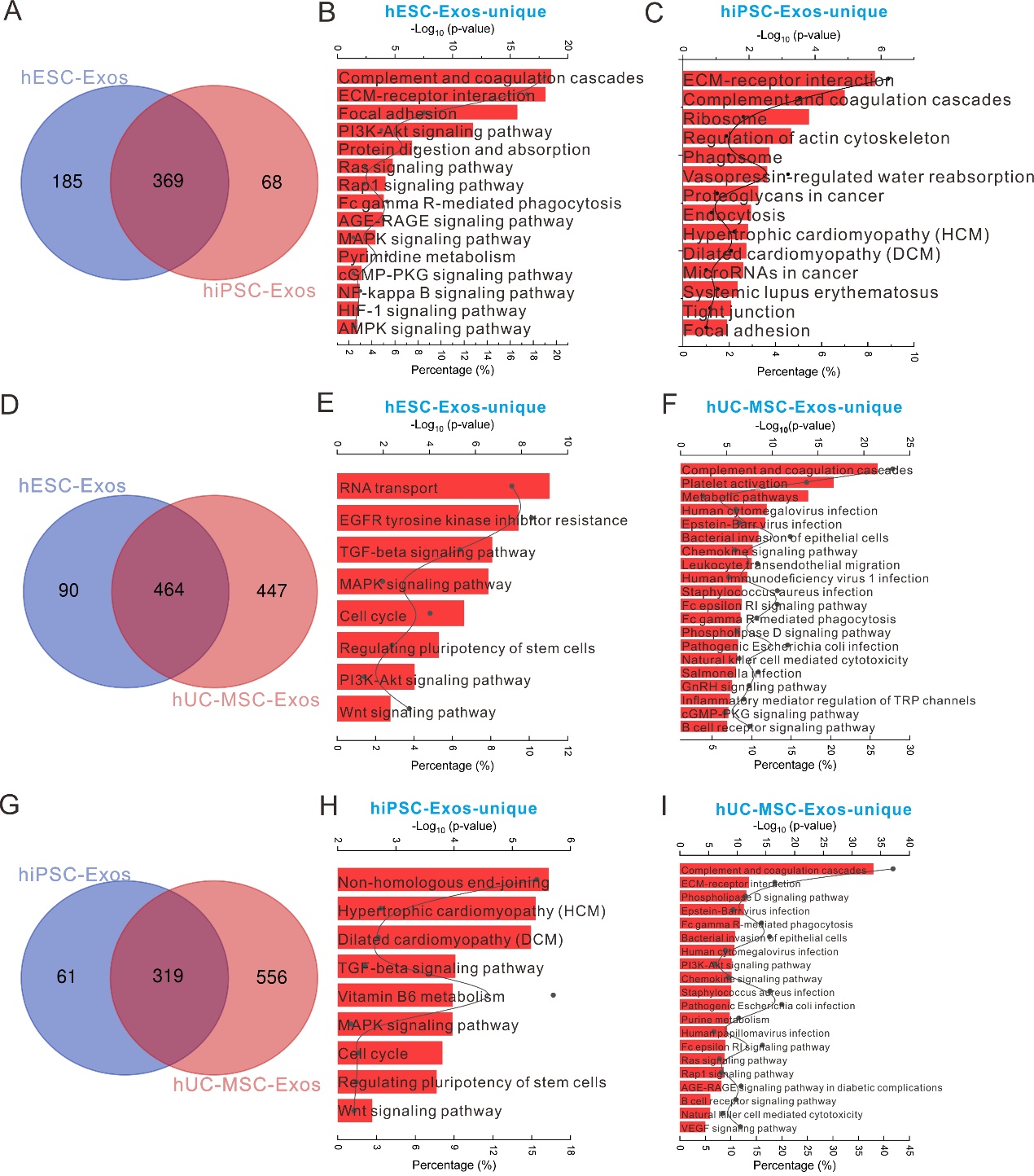
**Fig. S4 KEGG analysis of unique proteins when performed pairwise bioinformatic analysis of exosome proteomics.** (A, D, G) Venn diagram of pairwise analysis of hESC-Exos *vs.* hiPSC-Exos, hESC-Exos *vs.* hUC-MSC-Exos, and hiPSC-Exos *vs.* hUC-MSC-Exos. (B-C) KEGG analysis of unique proteins of hESC-Exos and hiPSC-Exos when performed their pairwise analysis. (E-F) KEGG analysis of unique proteins of hESC-Exos and hUC-MSC-Exos when performed their pairwise analysis. (H-I) KEGG analysis of unique proteins of hiPSC-Exos and hUC-MSC-Exos when performed their pairwise analysis. The red bar and black dot represent -log_10_ (p-value) and the proportion of candidate genes in the total pathway-related genes.


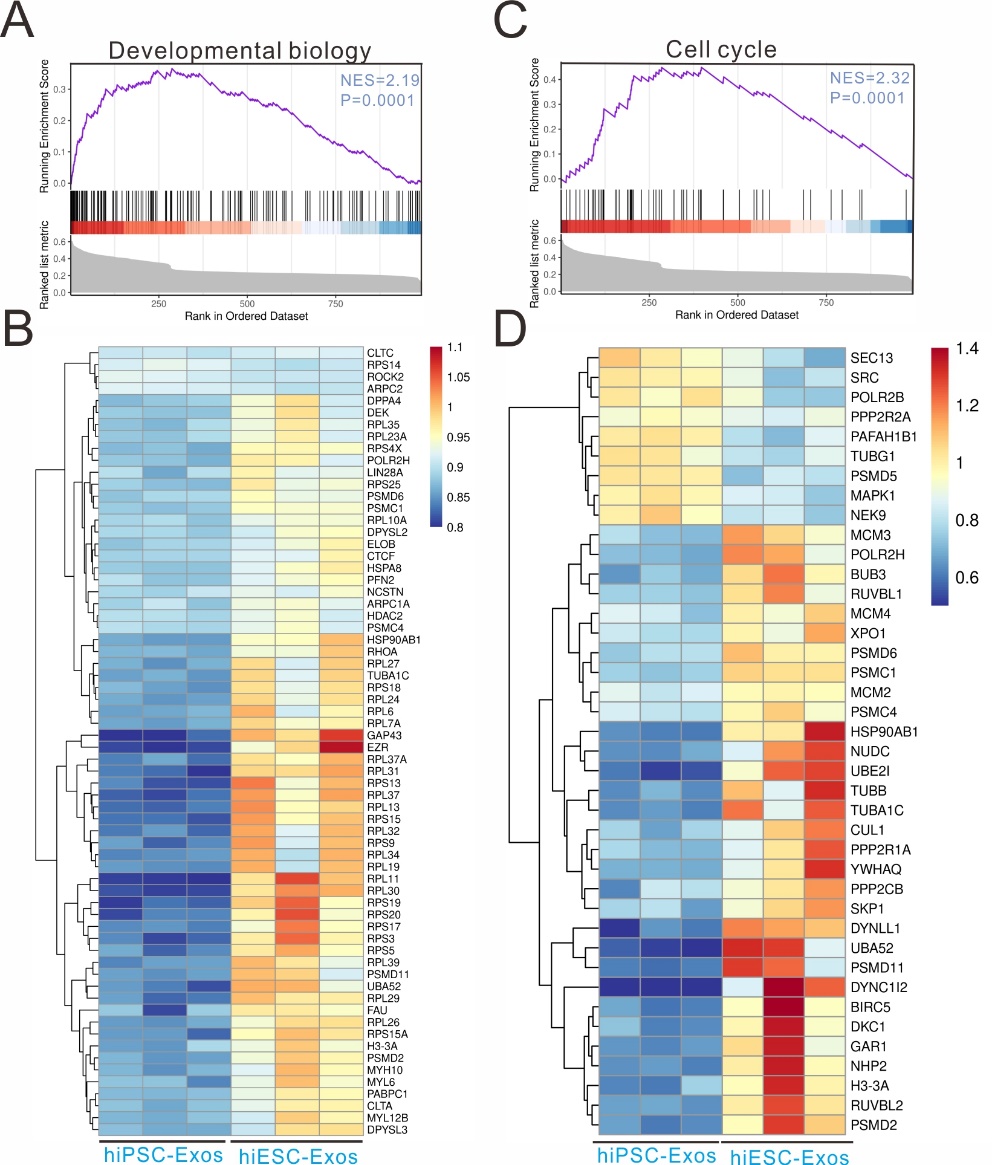
**Fig. S5 GSEA analysis of shared proteins between hESC-Exos and hiPSC-Exos.** (A) GESA prolife of shared proteins between hESC-Exos and hiPSC-Exos against the signature genes of developmental biology. (B) Heatmap showing relative expression levels of shared proteins enriched in developmental biology GSEA plot. (C) GESA prolife of shared proteins of hESC-Exos and hiPSC-Exos against the signature genes of cell cycle. (D) Heatmap showing relative expression levels of shared proteins enriched in cell cycle GSEA plot.


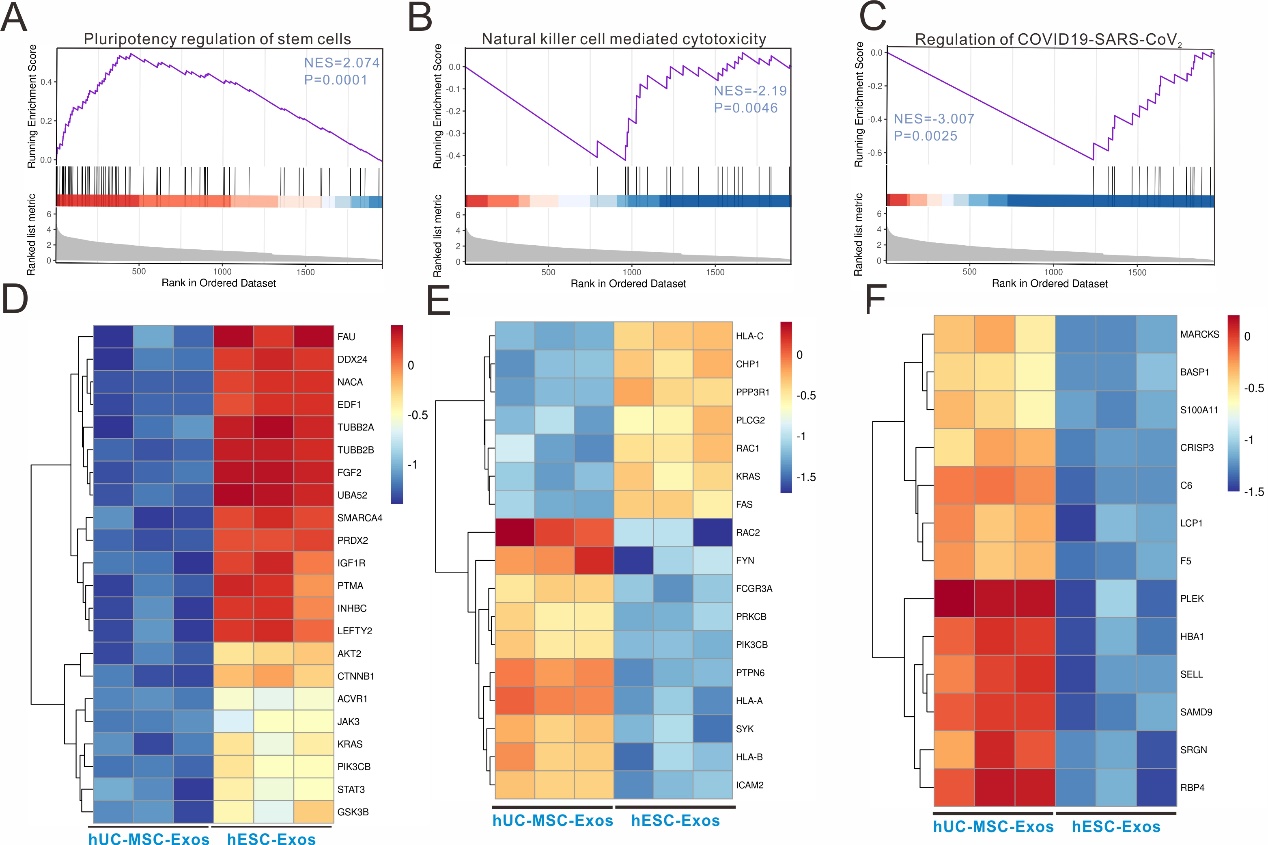


**Fig. S6 GSEA analysis of shared proteins between hESC-Exos and hUC-MSC-Exos.** (A) GSEA prolife of shared proteins against the signature genes of pluripotency regulation of stem cells. (B) GSEA prolife of shared proteins against the signature genes of natural killer cell-mediated cytotoxicity. (C) GSEA prolife of shared proteins against the signature genes of regulation of COVID19-SARS-CoV_2_. (D) Heatmap showing relative expression levels of shared proteins enriched in the GSEA plot (A). (E) Heatmap showing relative expression levels of shared proteins enriched in the GSEA plot (B). (F) Heatmap showing relative expression levels of shared proteins enriched in the GSEA plot (C).

**
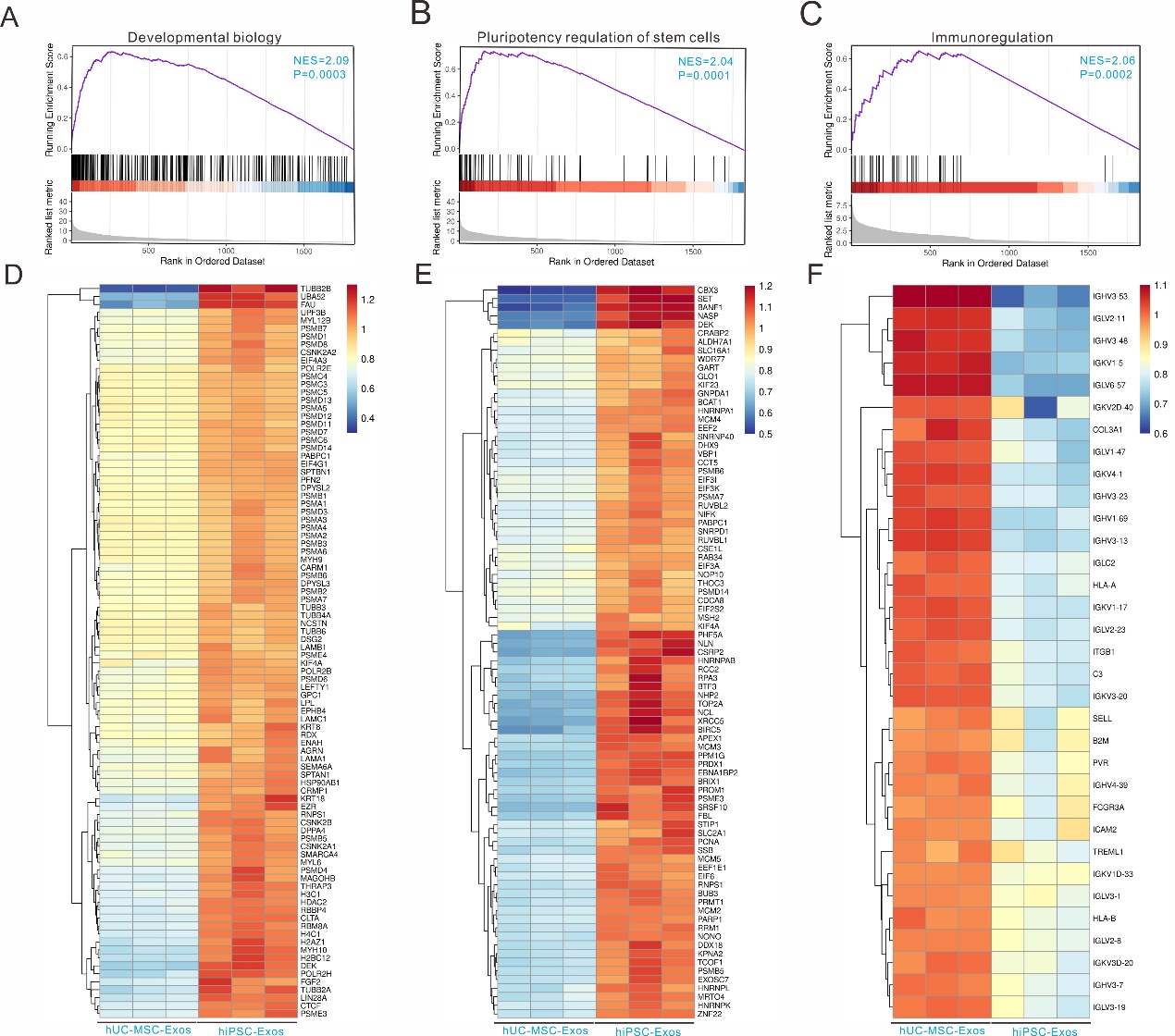
Fig. S7 GSEA analysis of shared proteins between hiPSC-Exos and hUC-MSC-Exos.** (A) GSEA prolife of shared proteins against the signature genes of developmental biology. (B) GSEA prolife of shared proteins against the signature genes of pluripotency regulation of stem cells. (C) GSEA prolife of shared proteins against the signature genes of immunoregulation. (D) Heatmap showing relative expression levels of shared proteins enriched in the GSEA plot (A). (E) Heatmap showing relative expression levels of shared proteins enriched in the GSEA plot (B). (F) Heatmap showing relative expression levels of shared proteins enriched in the GSEA plot (C).


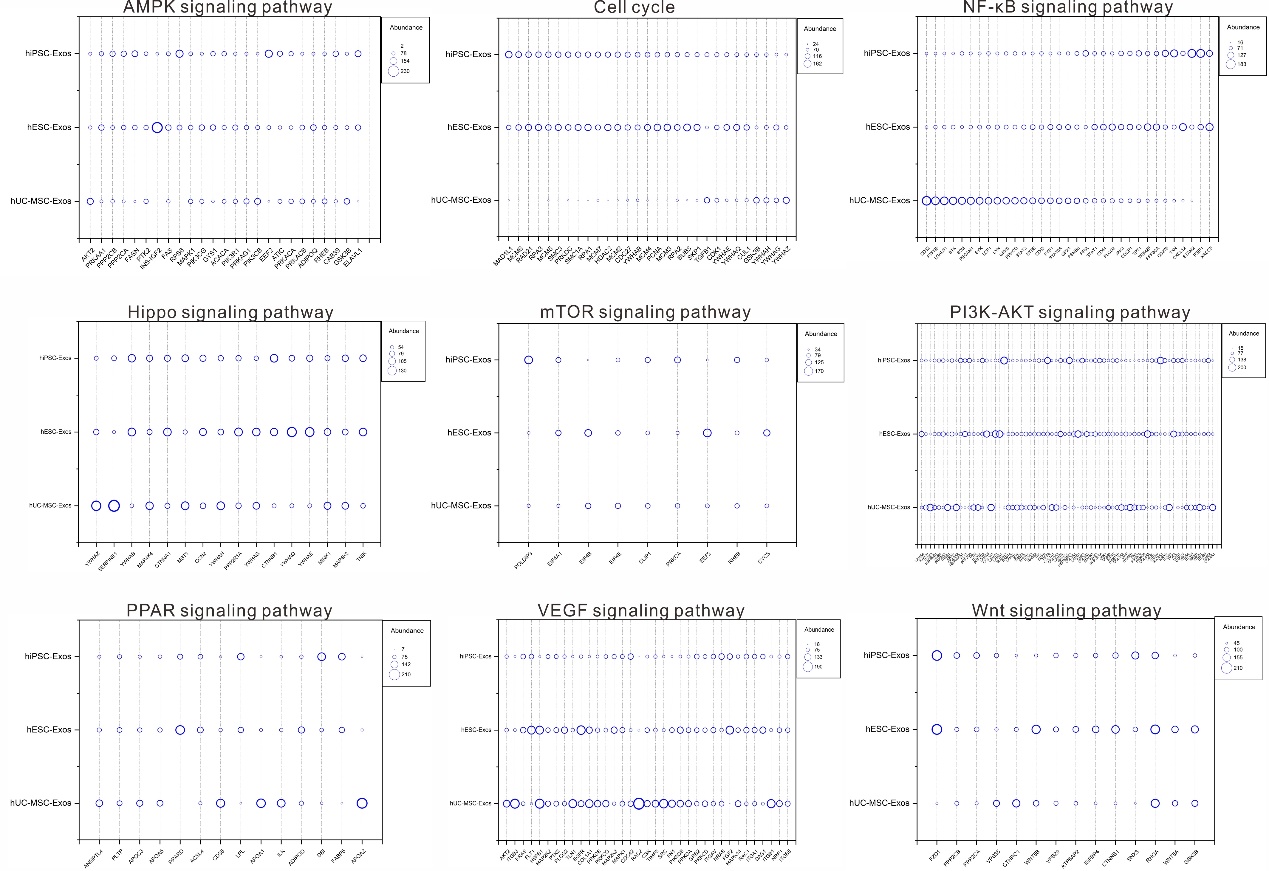
**Fig. S8 The protein abundance of shared proteins among three exosomes in different signaling pathways. Bubble size represents protein abundance.**


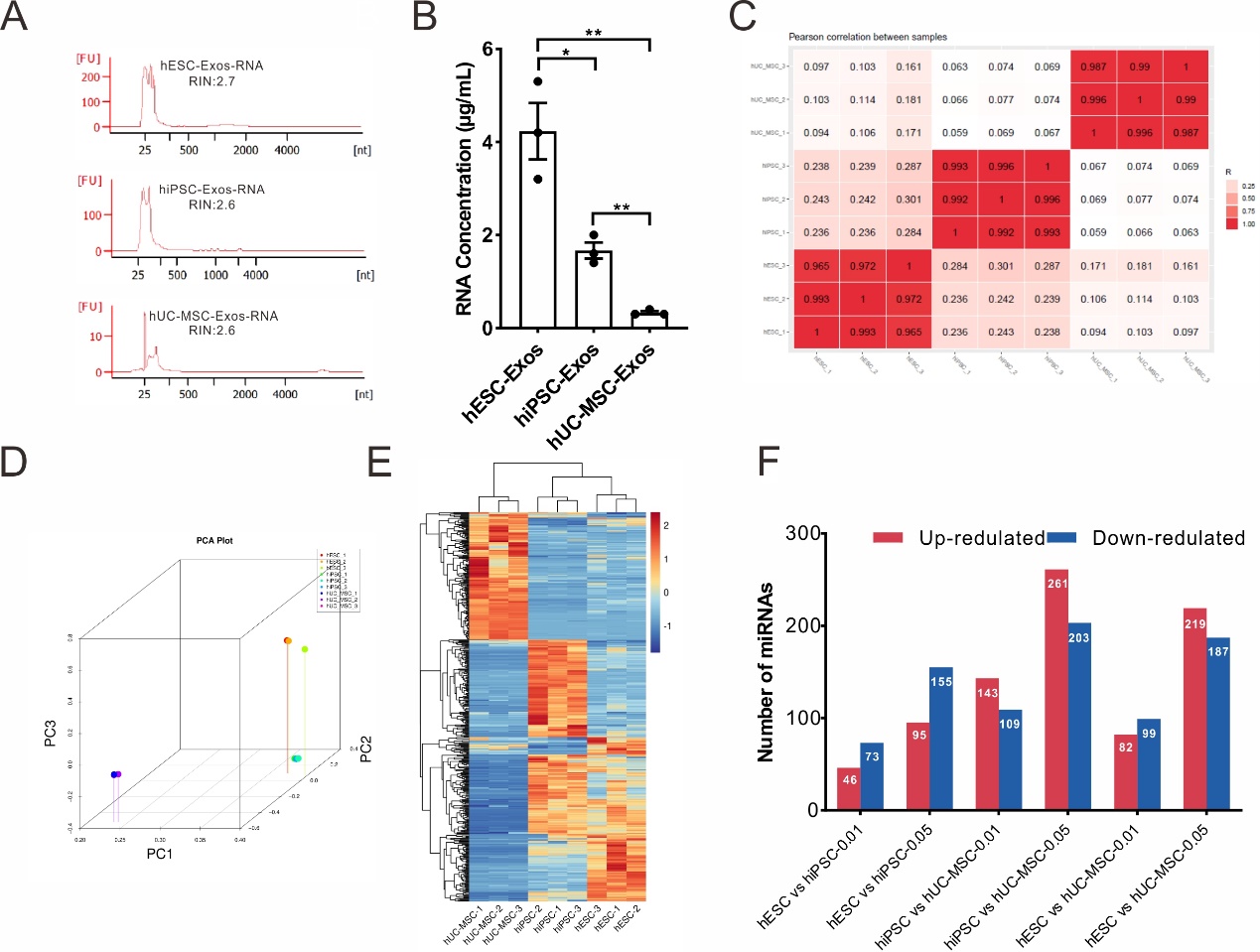
**Fig. S9 RNA quality inspection and abundance of three exosomes.** (A) Assessment of RNA integrity. (B) NanoDrop recorded the RNA concentration of three exosomes with selfsame volume (n = 3 samples per group). All statistical data are represented as the mean ± standard deviation. ^*^*P*＜0.05; ^**^*P*＜0.01. (C) Pearson correlation between the three exosome samples. (D) PCA map of isolated RNA from the three exosomes. (E) Heatmap of miRNA expression among the three exosomes. (F) The number of upregulated and downregulated miRNAs between any two exosomes at *P*＜0.05 or 0.01.


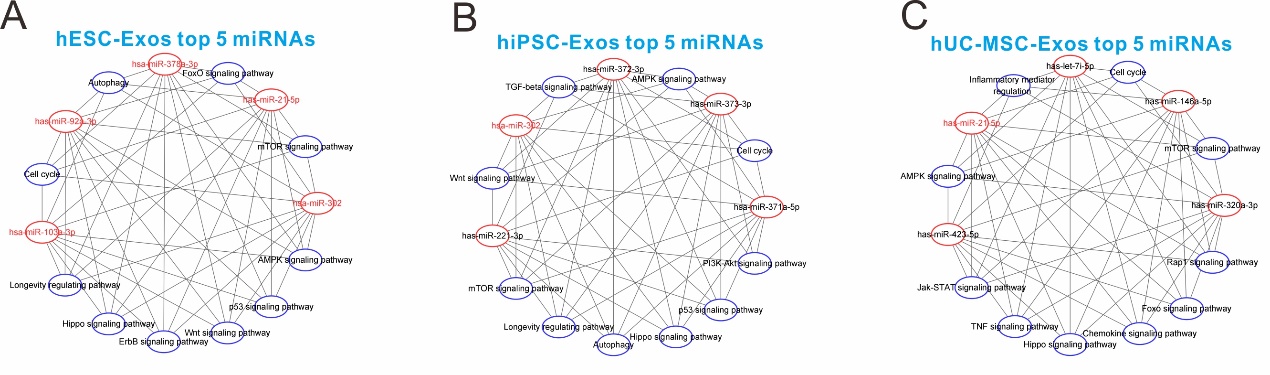
**Fig. S10 The crosstalk between top 5 miRNAs in each exosome and canonical signaling pathways.**


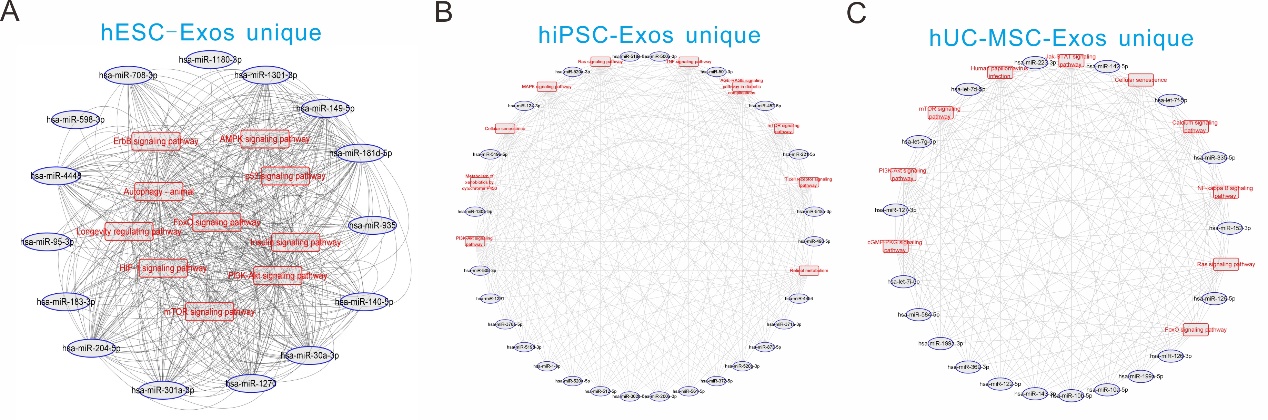


**Fig. S11 The crosstalk between the unique miRNAs in each exosome and canonical signaling pathways.**

**Reference**

1. Bi Y, Guo X, Zhang M, Zhu K, Shi C, Fan B, Wu Y, Yang Z, Ji G (2021) Bone marrow derived-mesenchymal stem cell improves diabetes-associated fatty liver via mitochondria transformation in mice. Stem Cell Res Ther 12 (1):602. doi:10.1186/s13287-021-02663-5

2. Dominici M, Le Blanc K, Mueller I, Slaper-Cortenbach I, Marini F, Krause D, Deans R, Keating A, Prockop D, Horwitz E (2006) Minimal criteria for defining multipotent mesenchymal stromal cells. The International Society for Cellular Therapy position statement. Cytotherapy 8 (4):315-317. doi:10.1080/14653240600855905

3. Gong L, Chen B, Zhang J, Sun Y, Yuan J, Niu X, Hu G, Chen Y, Xie Z, Deng Z, Li Q, Wang Y (2020) Human ESC-sEVs alleviate age-related bone loss by rejuvenating senescent bone marrow-derived mesenchymal stem cells. Journal of extracellular vesicles 9 (1):1800971. doi:10.1080/20013078.2020.1800971

**Table S1. Antibodies used in this study.**

| **Primary Antibody** | **Company (Catalogue NO.)** | **Dilution** |
| --- | --- | --- |
| TSG101 | Abcam (Cat^#^ ab30871) | 1:1000 for WB |
| CD63 | Abcam (Cat^#^ ab217345) | 1:1000 for WB |
| Calnexin | Abcam (Cat^#^ ab133615) | 1:2000 for WB |
| GAPDH | Abcam (Cat^#^ ab8245) | 1:5000 for WB |
| MCM5 | Abcam (Cat^#^ ab75975) | 1:500 for WB |
| PCNA | Abcam (Cat^#^ ab29) | 1:1000 for WB |
| CDK1 | Abcam (Cat^#^ ab201008) | 1:1000 for WB |
| PRKAA1 | Abcam (Cat^#^ ab32047) | 1:1000 for WB |
| SYK | Abcam (Cat^#^ ab40781) | 1:1000 for WB |
| BTK | Abcam (Cat^#^ ab208937) | 1:1000 for WB |
| Wnt5 | Abcam (Cat^#^ ab229200) | 1:1000 for WB |
| RHEB | Abcam (Cat^#^ ab92313) | 1:1000 for WB |
| GSK3B | Abcam (Cat^#^ ab93926) | 1:1000 for WB |
| EGFR | Abcam (Cat^#^ ab52894) | 1:1000 for WB |
| ICAM2 | Abcam (Cat^#^ 189463) | 1:1000 for WB |
| HSP70 | Abcam(Cat^#^ ab2787) | 1:1000 for WB |

**Table S2. miRNA primers used in this study (Poly A tailing).**

| miRNA | Forward primer (5’-3’) |
| --- | --- |
| has-miR-302 | TAAGTGCTTCCATGTTTTAGTAG |
| has-miR-92a-3p | TATTGCACTTGTCCCGGCCTGT |
| has-miR-21-5p | TAGCTTATCAGACTGATGTTGA |
| has-miR-378a-3p | ACTGGACTTGGAGTCAGAAGGC |
| has-miR-103a-3p | AGCAGCATTGTACAGGGCTATGA |
| has-miR-372-3p | AAAGTGCTGCGACATTTGAGCGT |
| has-miR-371a-5p | ACTCAAACTGTGGGGGCACTTT |
| has-miR-221-3p | AGCTACATTGTCTGCTGGGTTTC |
| has-miR-373-3p | GAAGTGCTTCGATTTTGGGGTGT |
| has-miR-146a-5p | TGAGAACTGAATTCCATGGGTT |
| has-miR-423-5p | AGCTCGGTCTGAGGCCCCTCAGT |
| has-miR-320a-3p | AAAAGCTGGGTTGAGAGGGCGA |
| has-let-7i-5p | TGAGGTAGTAGTTTGTGCTGTT |
